# Fano Effect and Quantum Entanglement in Hybrid Semiconductor Quantum Dot-Metal Nanoparticle System

**DOI:** 10.3390/s17061445

**Published:** 2017-06-20

**Authors:** Yong He, Ka-Di Zhu

**Affiliations:** 1 School of Mathematics and Physics, Changzhou University, Changzhou 213164, China; 2 Key Laboratory of Artificial Structures and Quantum Control (Ministry of Education), Department of Physics, Shanghai Jiao Tong University, 800 Dong Chuan Road, Shanghai 200240, China; zhukadi@sjtu.edu.cn

**Keywords:** fano effect, quantum entanglement, semiconductor quantum dot, metal nanoparticle

## Abstract

In this paper, we review the investigation for the light-matter interaction between surface plasmon field in metal nanoparticle (MNP) and the excitons in semiconductor quantum dots (SQDs) in hybrid SQD-MNP system under the full quantum description. The exciton-plasmon interaction gives rise to the modified decay rate and the exciton energy shift which are related to the exciton energy by using a quantum transformation method. We illustrate the responses of the hybrid SQD-MNP system to external field, and reveal Fano effect shown in the absorption spectrum. We demonstrate quantum entanglement between two SQD mediated by surface plasmon field. In the absence of a laser field, concurrence of quantum entanglement will disappear after a few ns. If the laser field is present, the steady states appear, so that quantum entanglement produced will reach a steady-state entanglement. Because one of all optical pathways to induce Fano effect refers to the generation of quantum entangled states, It is shown that the concurrence of quantum entanglement can be obtained by observation for Fano effect. In a hybrid system including two MNP and a SQD, because the two Fano quantum interference processes share a segment of all optical pathways, there is correlation between the Fano effects of the two MNP. The investigations for the light-matter interaction in hybrid SQD-MNP system can pave the way for the development of the optical processing devices and quantum information based on the exciton-plasmon interaction.

## 1. Introduction

Advances in modern nanoscience have allowed for the construction of various nanostructures such as metal nanoparticles (MNPs) and semiconductor quantum dots (SQDs) for their applications in photonics and optoelectronics. Studies of these nanostructures are essential for further development of nanotechnology. The optical properties of these nanostructures are very interesting topics, which can be exploited to design various optical processing devices. In a hybrid nanocrystal complex composed of SQDs and MNPs, due to the exciton-plasmon interaction several interesting phenomena, such as energy transfer [1], local field enhancement [2], and thermal effects [3], have been explored. These phenomena depend strongly on these particles geometry and their coupling. The modified decay rate and the shifted exciton frequency of SQD are also reported in the presence of MNP [4,5]. They are related to the distance between SQD and MNP.

Metal nanowire can be excited to produce surface plasmon polaritons [6] which are propagating charge density waves with associated strong enhanced electromagnetic field [7,8,9,10]. Surface plasmon field can excite a SQD from its ground state to the excited state. To study the light-matter interaction between surface plasmon field in MNPs and the excitons in SQDs, there are two descriptions, i.e., semiclassical description and quantum description. Semiclassical description is that the exciton is described in the quantum framework while the description of surface plasmon field is within the classical electromagnetic dynamics [11,12,13]. In quantum description, however, surface plasmon field has been quantized for showing quantum effects [14,15,16,17]. Surface plasmon field in MNP can be considered as a multi-modes field, the Hamiltonian can be written as $H_{P}=\sum_{k}\hbar \omega_{k}a_{k}^{+}a_{k}$ [14,15,18,19], where $\omega_{k}$ is the frequency of mode *k*, $a_{k}^{+}$ ($a_{k}$) is the creation (annihilation) operator of mode *k*. The quantization of surface plasmon field opens up a new frontier in the study of the fundamental physics of surface plasmons [20]. Quantum description of the exciton-plasmon interaction paves the way for various applications such as single-photon transistors [21], quantum information processing [22], etc.

Fano effect appears in the energy absorption spectrum in a hybrid molecule consisting of SQDs and MNPs. One of the main features of the Fano effect is its asymmetric line profile showed in optical absorption spectrum [23]. It is well known that because of the exciton-plasmon interaction the energy absorption of a MNP shows the Fano effect which originates from a Fano interference process between two competing optical pathways [24]. Recently, Zhang and Govorov have demonstrated that in a simple hybrid system including a SQD and a MNP the Fano effect from quantum description differs both qualitatively and quantitatively from that of semiclassical description, especially in the strong field regime [15]. This implies that quantum description for the exciton-plasmon interaction can reveal more novel optical properties that may be applied in optical processing devices in the future. However, for some more complex systems including a few SQDs and MNPs, the quantum description needs to be further developed to reveal the quantum nature of the exciton-plasmon interaction.

In this paper, we will briefly review our investigation for optical properties and quantum entanglement of the coupled SQD-MNP system based on quantum description for the exciton-plasmon interaction. Cavity quantum electrodynamics (QED) as a quantum optics toolbox provides a full quantum mechanics description of the coupled SQD-MNP system. Under quantum description we proposed a quantum transformation method that is suitable for the coupling of excitons to surface plasmon field with large decay rate. The quantum transformation is used to treat master equation of the entire system for obtaining an effective Hamiltonian in SQD’ subsystem. In this way, we investigated three sorts of the coupled SQD-MNP systems, i.e., a hybrid system including a SQD and a MNP [25], a hybrid system including two SQDs and a MNP [26,27], and a hybrid system including a SQD and two MNPs [28]. The quantum transformation method is advantaged for the investigation of the hybrid systems including a few nanoparticles. Recently, Hayati etc. have developed an efficient self-consistent field method based on the discrete dipole approximation for obtaining the optical response of some large hybrid networks of SQDs and MNPs [29]. This review is organized as follows. We first introduce the exciton-plasmon interaction under quantum description and a quantum transformation method in Section 2, and then show the optical properties of a hybrid system including a SQD and a MNP in Section 3. In Section 4, we demonstrate the coupling of two SQDs induced by a MNP and optical detection of quantum entanglement between the two SQDs. In Section 5, we show the correlation between two Fano interferences in a hybrid system including two MNPs and a SQD. We conclude this review in Section 6.

## 2. Theory

**Figure 1:**
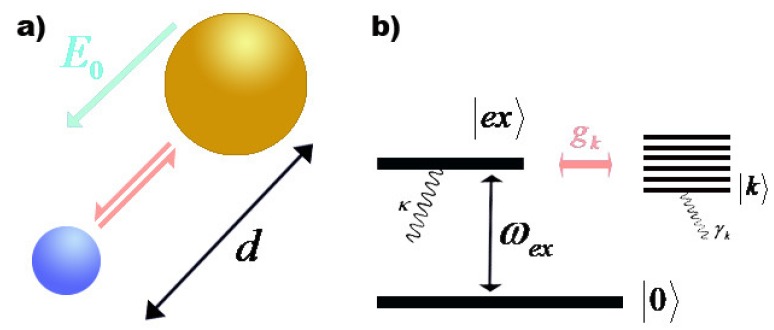
(**a**) Schematic illustration of the hybrid SQD-MNP system; (**b**) The energy level diagram.

To study the exciton-plasmon interaction, we consider a simple model which includes a spherical MNP with radius *R* coupling to a spherical SQD with radius *r* consisted of the electronic ground state $|0\rangle$ and the first excited state $|ex\rangle$ in the presence of an external field $E=E_{0}e^{-i\omega t}+c.c.$. The center-to-center distance between them is *d*. The entire system is embedded in a dielectric medium with constant permittivity $\varepsilon_{0}$, as illustrated in Figure 1a.

The Hamiltonian of an individual SQD can be expressed as (ℏ=1)

(1) $$H_{SQD}=\omega_{ex}\sigma_{z},$$

where $\sigma_{z}=\left(|ex\rangle \langle ex|-|0\rangle \langle 0|\right)/2$. Surface plasmon field in the MNP is induced by the external field and the dipole of the SQD. It can be considered as a multiple-modes field. After the second quantization of surface plasmon field, the Hamiltonian can be written [18,19]

(2) $$H_{P}=\sum_{k}\omega_{k}a_{k}^{+}a_{k},$$

where $\omega_{k}$ is the frequency of mode *k*, $a_{k}^{+}$ ($a_{k}$) is the creation (annihilation) operator of mode *k*. The coupling between the SQD and the MNP is the result of the energy transfer between the exciton and the plasmon field. In cavity quantum electrodynamics, the energy transfer is always described by the Jaynes-Cummings model under the rotating-wave approximation. So, the interaction Hamiltonian between the SQD and MNP can be written as [14,15,30]

(3) $$H_{int}=-\sum_{k}\left(g_{k}a_{k}\sigma_{+}+h.c.\right),$$

where $\sigma_{+}=\left|ex><0\right|$, $g_{k}$ is the coupling constant between the exciton and the mode *k*. On the right-hand side of the above equation, the term $a_{k}\sigma_{+}$ corresponds to the absorption of a photon and the excitation of the exciton from the ground state to the excited state, and vice versa. The two processes describe the energy transfer between the exciton and the plasmon field. If the hybrid system is driven by the strong laser field with frequency $\omega_{s}$, the driving Hamiltonian is given by

(4) $$H_{D}=-\sum_{k}\left(\mu \sigma_{+}+\mu_{k}^{*}a_{k}^{+}\right)E_{s}e^{-i\omega_{s}t}+h.c.,$$

where μ ($\mu_{k}$) is the dipole moment between the ground state and exciton-excited state in SQD (plasmon-excited states in MNP) [15].

In a rotating frame at the external field frequency ω, the entire system Hamiltonian is given by

(5) $$H=\left(\omega_{ex}-\omega \right)\sigma_{z}+\sum_{k}(\omega_{k}-\omega )a_{k}^{+}a_{k}-\sum_{k}\left[g_{k}a_{k}\sigma_{+}+\left(\mu \sigma_{+}+\mu_{k}^{*}a_{k}^{+}\right)E_{0}+h.c.\right]$$

The full quantum dynamics of the coupled nanosystem can be derived from the following master equation for the density operator

(6) $$\partial_{t}\rho =-i\left[H,\rho \right]+\varsigma_{S}+\varsigma_{P},$$

with the Liouvillian terms, $\varsigma_{S}=\left(\kappa /2\right)\times \left(2\sigma \rho \sigma_{+}-\rho \sigma_{+}\sigma -\sigma_{+}\sigma \rho \right)$ describes the decay of SQD to Markovian reservoirs, κ is the exciton radiative decay rate in SQD, $\varsigma_{P}=\sum_{i}\left(\gamma_{k}/2\right)\times \left(2a_{k}\rho a_{k}^{+}-\rho a_{k}^{+}a_{k}-a_{k}^{+}a_{k}\rho \right)$ describes the relaxation of plasmonic mode *k* with decay rate $\gamma_{k}$.

Based on Equation (6), the expectation values $<a_{k}>$, <σ> and $<\sigma_{z}>$ satisfy the following equations,

(7) $$i\partial_{t}\left[\sum_{k}\pi_{k}\left(\omega \right)<a_{k}>\right]=-i\sum_{k}g_{k}<a_{k}>-\sum_{k}\pi_{k}\left(\omega \right)\left(g_{k}^{*}<\sigma >+\mu_{k}^{*}E_{0}\right),$$

(8) $$i\partial_{t}<\sigma >=\left(\omega_{ex}-\omega -i\kappa /2\right)<\sigma >+2<\sigma_{z}>\left(\sum_{k}g_{k}<a_{k}>+\mu E_{0}\right),$$

(9) $$i\partial_{t}<\sigma_{z}>=-i\kappa \left(<\sigma_{z}>+1/2\right)-\left[<\sigma_{+}>\left(\sum_{k}g_{k}<a_{k}>+\mu E_{0}\right)-c.c.\right],$$

where $\pi_{k}\left(\omega \right)=2g_{k}/\left[2\left(\omega_{k}-\omega \right)i+\gamma_{k}\right]$, and <σ> represents the probability amplitude of the transition from the ground state to the excited state. Because the above equations can not be solved accurately, we have to resort to the steady state limit method.

In the steady state limit, using Equation (7) we have

(10) $$\sum_{k}g_{k}<a_{k}>=i\sum_{k}\pi_{k}\left(\omega \right)\left(g_{k}^{*}<\sigma >+\mu_{k}^{*}E_{0}\right).$$

With respect to the quantization of the electric field $E_{MNP}$ induced by the MNP, the electric field operator can be written as $\hat{E}\left(d\right)$. It can be split into two contributions ${\hat{E}}^{+}\left(d\right)+{\hat{E}}^{-}\left(d\right)$ evolving with positive and negative frequencies, and $\mu {\hat{E}}^{+}\left(d\right)=\sum_{k}{g_{k}a}_{k}$ [31], where $<{\hat{E}}^{+}\left(d\right)>=E_{MNP}$, and $E_{MNP}=E_{loc}+E_{pol}$, $E_{loc}=\left(s_{\alpha }\gamma R^{3}E_{0}\right)/\left(\varepsilon_{eff1}d^{3}\right)$ comes from surface charges of the MNP induced by the external field. $E_{pol}=\left(\mu s_{\alpha }^{2}\gamma R^{3}P^{+}\right)/\left(\varepsilon_{eff1}\varepsilon_{eff2}d^{6}\right)$ is the effective electric field produced by the dipole polarization in the MNP induced by the effective dipole of the SQD, $P^{+}=\mu <\sigma >$, $\varepsilon_{eff1}=\left(\varepsilon_{S}+2\varepsilon_{0}\right)/\left(3\varepsilon_{0}\right)$, $\varepsilon_{eff2}=\left(\varepsilon_{S}+2\varepsilon_{0}\right)/3$, $\gamma =\left[\varepsilon_{M}\left(\omega \right)-\varepsilon_{0}\right]/\left[\varepsilon_{M}\left(\omega \right)+2\varepsilon_{0}\right]$, $\varepsilon_{S}$ and $\varepsilon_{M}\left(\omega \right)$ are the dielectric constants of the SQD and the MNP, respectively. $s_{\alpha }=2\left(-1\right)$ for the exciton-dipole orientation parallel (perpendicular to) the axis of the hybrid nanocrystal complex of the SQD and the MNP [11,12]. Therefore, using Equation (10) we have

(11) $$i\sum_{k}\pi_{k}\left(\omega \right)\left(g_{k}^{*}<\sigma >+\mu_{k}^{*}E_{0}\right)=\frac{\mu^{2}s_{\alpha }^{2}\gamma R^{3}<\sigma >}{\varepsilon_{eff1}\varepsilon_{eff2}d^{6}}+\frac{\mu s_{\alpha }\gamma_{1}R^{3}E_{0}}{\varepsilon_{eff1}d^{3}}.$$

Further, $\sum_{k}\mu_{k}^{*}\pi_{k}\left(\omega \right)=-i\mu C\left(\omega \right)$, $\sum_{k}g_{k}^{*}\pi_{k}\left(\omega \right)=-iG\left(\omega \right)$, where $C\left(\omega \right)=\left(s_{\alpha }\gamma R^{3}\right)/\left(\varepsilon_{eff1}d^{3}\right)$, $G\left(\omega \right)=\left(\mu^{2}s_{\alpha }^{2}\gamma R^{3}\right)/\left(\hbar \varepsilon_{eff1}\varepsilon_{eff2}d^{6}\right)$. Since ω is arbitrary, we have

(12) $$g_{k}\equiv \frac{\mu s_{\alpha }}{\hbar \varepsilon_{eff2}d^{3}}\mu_{k},\sum_{k}\frac{|\mu_{k}{|}^{2}}{\left(\omega_{k}-\omega \right)-i\gamma_{k}/2}\equiv \hbar \varepsilon_{0}\gamma R^{3}.$$

The above equations show the quantum-semiclassical relation. If only one plasmon mode in the MNP is considered, the expression of the coupled strength between the SQD and the MNP can be obtained by the derivation, as illustrated in Ref. [32]. Considering the multiple-modes field, Zhang and Govorov establish the quantum-semiclassical relation for revealing the optical properties of the coupled SQD-MNP system under the quantum description [15].

We try to treat the density operator for obtaining the reduced density operator of the SQD. Firstly, we take a time-independent unity transformation $e^{is}$ on the density operator ρ, where $s=\sum_{k}{[\pi_{k}(\omega }_{ex}{)a_{k}\sigma_{+}+\pi_{k}^{*}(\omega }_{ex})a_{k}^{+}\sigma ]$, so that $\tilde{\rho }=e^{is}\rho e^{-is}$.

(13) $$\partial_{t}\tilde{\rho }=-i\left[e^{is}He^{-is},\tilde{\rho }\right]+e^{is}\varsigma_{S}e^{-is}+e^{is}\varsigma_{p}e^{-is}.$$

For the mathematical expansions of $e^{is}He^{-is}$, $e^{is}\varsigma_{S}e^{-is}$, $e^{is}\varsigma_{p}e^{-is}$, we can neglect the terms of order $O(g_{k}^{3})$ and higher. In order to obtain the reduced density operator of the SQD $\rho_{S}=Tr_{p}\left[\tilde{\rho }\right]$, we assume that the multi-mode plasmon field can be consider as a thermal reservoir and the reservoir variables are distributed in the uncorrelated thermal equilibrium mixture of states [33], $<a_{k}^{+}a_{l}>=n_{k}\delta_{kl}$, where the thermal average boson number ${\left(n_{k}\right)}^{-1}=exp\left[\left(\hbar \omega_{k}\right)/\left(k_{B}T\right)\right]-1$, $k_{B}$ is the Boltzmann constant, and *T* is the temperature. At room temperature $T\ll \left(\hbar \omega_{k}\right)/k_{B}$ ($\hbar \omega_{k}∼eV$), so $n_{k}\ll 1$.

In the subsystem of the SQD, the master equation can be written as

(14) $$\partial_{t}\rho_{S}=-i\left[H_{S},\rho_{S}\right]+\zeta_{S},$$

where,

(15) $$H_{S}=\left(\omega_{ex}^{0}-\omega \right)\sigma_{z}-\left(\mu_{0}E_{0}\sigma_{+}+h.c.\right),$$

(16) $$\zeta_{S}=\left(\kappa_{0}/2\right)\times \left(2\sigma \rho \sigma_{+}-\rho \sigma_{+}\sigma -\sigma_{+}\sigma \rho \right),$$

where $\omega_{ex}^{0}=\omega_{ex}-G_{R}\left(\omega_{ex}\right)$, $\kappa_{0}=\kappa +2G_{I}\left(\omega_{ex}\right)$, $\mu_{0}{=\mu [1+C(\omega }_{ex})]$, $G_{R}\left(\omega_{ex}\right)$ and $G_{I}\left(\omega_{ex}\right)$ are the real and imaginary parts of $G(\omega_{ex})$, respectively. We note that the exciton energy shift and the reduced lifetime of the SQD are related to the exciton energy. $\Gamma_{nr}=2G_{I}\left(\omega_{ex}\right)$ represents the non-radiative decay rate that can be decomposed into different contributions for each plasmon mode, i.e., $\Gamma_{nr}=\sum_{k}{\left|g_{k}\right|}^{2}\gamma_{k}/\left[{\left(\omega_{k}-\omega_{ex}\right)}^{2}+\gamma_{k}^{2}/4\right]$ [14]. We can see that the transformation treatment can reveal the optical phenomena induced by the plasmon field which have been reported experimentally. Another treatment approach to obtain the reduced density operator of the SQD can be implemented by using the effective time evolution superoperator in the Heisenberg picture [34]. In a two-level system driven by an external force, the approach can also be used to obtain the effective Hamiltonian [35].

**Figure 2:**
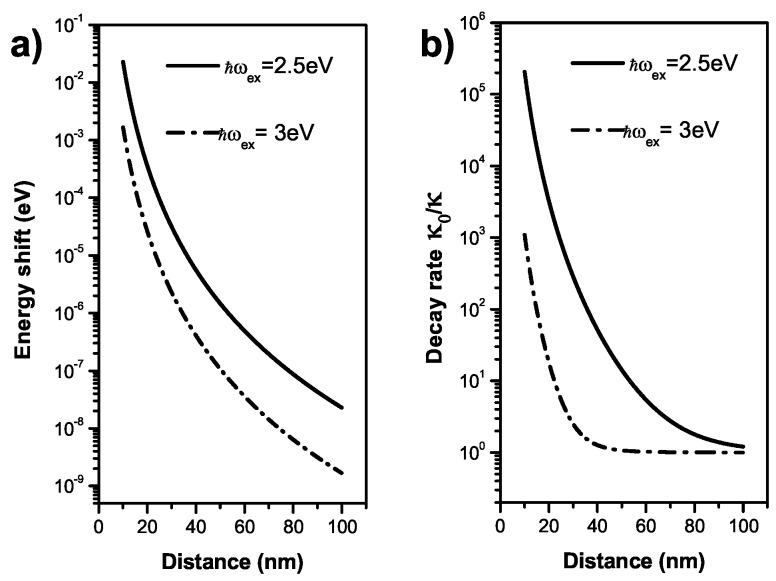
(**a**) The energy shift; (**b**) the modified decay rate as a function of the distance for $\hbar \omega_{ex}\;=\;2.5,3\;$eV.

Figure 2 shows the energy shift, the modified decay rate as a function of the distance for a given exciton energy. Here, we consider a Au nanoparticle with radius R=10 nm. Its dielectric constant is ${\varepsilon_{M}\left(\omega \right)=\varepsilon }_{b}-\omega_{p}^{2}/\left[\omega \left(\omega +i\eta \right)\right]$ with $\varepsilon_{b}=9.5$, $\hbar \omega_{p}=9$ eV, ℏη=0.07 eV [36]. And $s_{\alpha }=2$. The dielectric constant of the background medium is $\varepsilon_{0}=1.8$ (water), and the SQD $\varepsilon_{S}=6$. For the decay rate and the dipole moment of the exciton, we take κ=1 GHz and $\mu =er_{0}$ with $r_{0}=0.65$ nm. Because the exciton energy are related to the generation of plasmon field, it has important influence on the exciton-plasmon interaction which causes the exciton energy shift. As shown in Figure 2a, the exciton energy shifts for the exciton energy 2.5 eV and 3 eV are different for a same distance. However, another obvious phenomenon is that the decay rate of the exciton increases as a result of the exciton-plasmon interaction. The non-radiative decay rate induced by the MNP is increasing with the decreasing distance, and it depends strongly on the exciton energy [3] as illustrated in Figure 2b. Especially for a short distance, the non-radiative decay rate is much larger than the radiative decay rate, which causes a pronounced decrease of the exciton lifetime. The modified decay rate have been reported by observing photoluminescence spectra [37,38].

## 3. Optical Response of a Hybrid Molecule Including a SQD and a MNP

We consider the response of the entire system to the presence of a laser field $E_{0}$. The optical Bloch equations of the SQD are given by

(17) $$\partial_{t}\rho_{22}=i\mu_{0}E_{0}\rho_{21}-i\mu_{0}^{*}E_{0}^{*}\rho_{21}^{*}-\kappa_{0}\rho_{22},$$

(18) $$\partial_{t}\rho_{21}=i\left(\omega_{ex}^{0}-\omega \right)\rho_{21}+i\mu_{0}^{*}E_{0}^{*}(2\rho_{22}-1)-\kappa_{0}\rho_{21}/2,$$

where $\rho_{22}=<ex\left|\rho_{S}\right|ex>$, $\rho_{11}=<0\left|\rho_{S}\right|0>$, $\rho_{21}=<ex\left|\rho_{S}\right|0>$, $\rho_{11}+\rho_{22}=1$. In the steady state limit, we set the left hand side of Equations (17) and (18) to zero.

(19) $$\rho_{22}=\frac{2Im[\mu_{0}^{*}E_{0}^{*}\rho_{21}^{*}]}{\kappa_{0}},$$

(20) $$\rho_{21}=\frac{\mu_{0}^{*}E_{0}^{*}(1-2\rho_{22})}{\left(\omega_{ex}^{0}-\omega \right)+i\kappa_{0}/2}.$$

For a weak laser field $\rho_{22}\ll 1$, so, $1-2\rho_{22}\approx 1$,

(21) $$\rho_{22}=\frac{|\mu_{0}E_{0}{|}^{2}}{{\left(\omega_{ex}^{0}-\omega \right)}^{2}+\kappa_{0}^{2}/4}.$$

For a strong laser field,

(22) $$\rho_{22}=\frac{|\mu_{0}E_{0}{|}^{2}}{{\left(\omega_{ex}^{0}-\omega \right)}^{2}+\kappa_{0}^{2}/4+2{\left|\mu_{0}E_{0}\right|}^{2}}.$$

The total energy absorption rate takes the from $Q_{tot}=Q_{S}+Q_{M}$, where $Q_{S}=\hbar \omega_{ex}\kappa \rho_{22}/2$, $Q_{M}=\sum_{k}\hbar \omega_{k}\gamma_{k}<a_{k}^{+}a_{k}>/2$. We combine Equations (10), (12) and (22) to obtain the equations

(23) $$Q_{M}\left(\Delta \right)=Q\frac{|\Delta +F_{R}{|}^{2}+F_{I}^{2}}{\Delta^{2}+1},Q_{S}\left(\Delta \right)=\frac{M^{2}{\left|E_{0}\right|}^{2}\hbar \omega_{ex}\kappa }{2(\Delta^{2}+1)},$$

where $Q=\sum_{k}(\omega_{k}\gamma_{k}|\mu_{k}{|}^{2}|E_{0}{|}^{2})/\left\{2\hbar \left[{\left(\omega_{k}-\omega \right)}^{2}+\gamma_{k}^{2}/4\right]\right\}$, $\Delta =\left(\omega_{ex}^{0}-\omega \right)/\sqrt{\kappa_{0}^{2}/4+2{\left|\mu_{0}E_{0}\right|}^{2}}$, $F_{R}=\left(\theta \mu_{R}\right)/\sqrt{\kappa_{0}^{2}/4+2{\left|\mu_{0}E_{0}\right|}^{2}}$, $F_{I}=\sqrt{{\left[\theta \mu_{I}-\kappa_{0}/2\right]}^{2}+2{\left|\mu_{0}E_{0}\right|}^{2}}/\sqrt{\kappa_{0}^{2}/4+2{\left|\mu_{0}E_{0}\right|}^{2}}$, $M=\sqrt{\mu_{R}^{2}+\mu_{I}^{2}}/\sqrt{\kappa_{0}^{2}/4+2{\left|\mu_{0}E_{0}\right|}^{2}}$, $\mu_{0}=\mu_{R}+i\mu_{I}$, $g_{k}=\theta \mu_{k}$, $\theta =\left(\mu s_{\alpha }\right)/\left(\hbar \varepsilon_{eff2}d^{3}\right)$ is a real number. Using Equation (12), we can obtain

(24) $$Q=\frac{\omega |E_{0}{|}^{2}R^{3}Im\left[\varepsilon_{M}\left(\omega \right)\right]}{6|\varepsilon_{eff1}{|}^{2}}.$$

**Figure 3:**
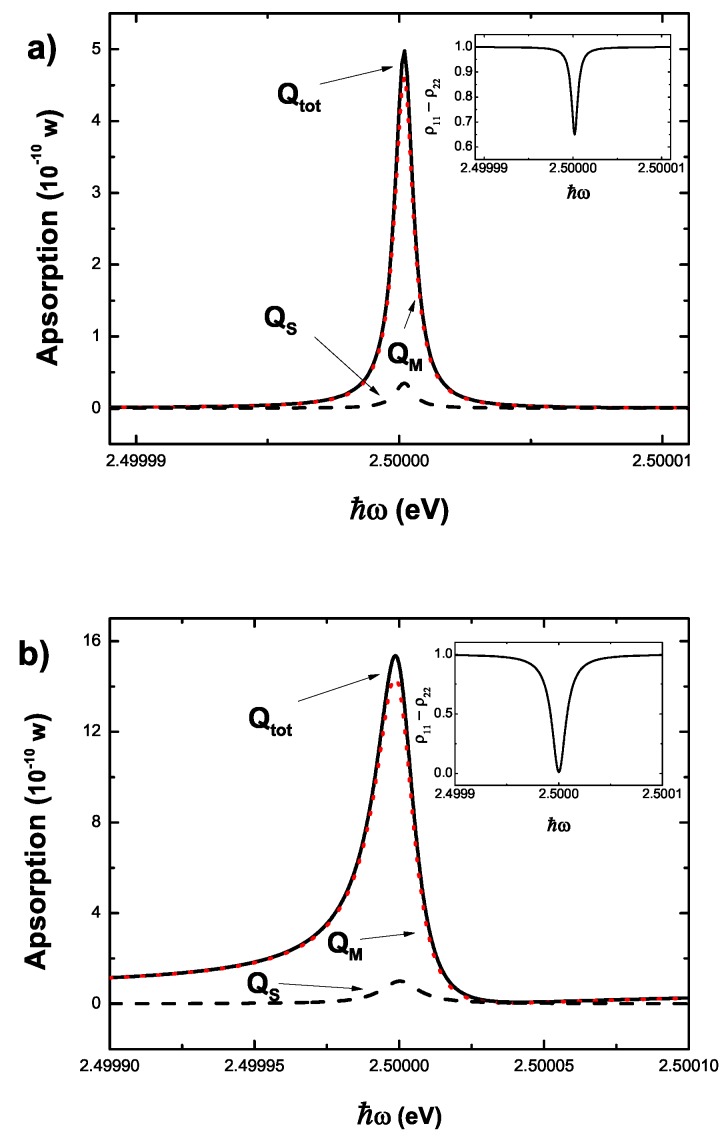
The energy absorption rate ($Q_{tot},Q_{M},Q_{S}$) as a function of the laser energy. (**a**) For a weak laser (the laser intensity is 1 W/cm${}^{2}$). Inset shows a population difference in the weak field regime; (**b**) For a strong laser (the laser intensity is 1000 W/cm${}^{2}$). Inset shows a population difference in the strong field regime.

To estimate the response of the system to a laser field, we take $\hbar \omega_{ex}=2.5$ eV and d=50 nm. Other parameters are the same as those of above section. Figure 3 shows the response of the entire system to a laser field. The total energy absorption rate includes two parts coming from the SQD $Q_{S}$ and the MNP $Q_{M}$, respectively. The Fano effect of the system is caused by $Q_{M}$ that has a Fano factor $F_{R}$ [23]. When the laser intensity is 1 W/cm${}^{2}$, a steep and symmetrical peak of the total energy absorption rate near ℏω=2.5 eV shows the absence of the Fano line profile. However, in the strong laser field (1000 W/cm${}^{2}$) the Fano effect appears as shown in Figure 3b. With the increasing laser intensity, the Fano effect becomes more pronounced because the Fano factor is a function of the laser intensity (see its expression). In Ref. [15], Zhang and Govorov compare Fano effect of the semiclassical theory with that of the quantum theory in the strong field, and show the difference between them. In a system consisting of two-level quantum emitter and one-dimensional coupled resonator waveguide, Zhou et al. illustrate a Fano-like line shape in the reflection spectrum of the resonator [39]. By controlling the quantum emitter, they show that Breit-Wigner-like line shape appears while Fano-like line shape disappears. This work can help to the study of Fano effects in the coupled emitter-resonator system.

Now, we consider a strong laser field $E_{s}$ and a weak laser field $E_{w}$ simultaneously presented in the system, i.e., $E_{0}=E_{s}+$$E_{w}e^{-i\delta t},$ $\omega_{s}=\omega ,$ $\omega_{w}=\omega +\delta ,$ $|E_{s}\left|\gg \right|E_{w}|.$ According to Equations (14)–(16), we have

(25) $$\partial_{t}p=\left[i\left(\omega -\omega_{ex}^{0}\right)-\kappa_{0}/2\right]p-i\mu \mu_{0}E_{0}w,$$

(26) $$\partial_{t}w=-\kappa_{0}\left(w+1\right)+4Im\left[\mu_{0}^{*}E_{0}^{*}p\right]/\mu ,$$

where $p=\mu_{12}\rho_{21},$ $w=\rho_{22}-\rho_{11}.$ In order to solve the above Equations, we make the ansatz [40]: $p=p_{0}+p_{+}e^{-i\delta t}+p_{-}e^{i\delta t}$, $w=w_{0}+w_{+}e^{-i\delta t}+w_{-}e^{i\delta t},$ and $|p_{0}\left|\gg \right|p_{+}\left|,\right|p_{-}|,$ $|w_{0}\left|\gg \right|w_{+}\left|,\right|w_{-}|$. Upon substituting these equations into Equations (25) and (26), we have

(27) $$0=\left[i\left(\omega -\omega_{ex}^{0}\right)-\kappa_{0}/2\right]p_{0}-i\mu \mu_{0}E_{s}w_{0},$$

(28) $$-i\delta p_{+}=\left[i\left(\omega -\omega_{ex}^{0}\right)-\kappa_{0}/2\right]p_{+}-i\mu \mu_{0}\left(E_{s}w_{+}+E_{w}w_{0}\right),$$

(29) $$i\delta p_{-}=\left[i\left(\omega -\omega_{ex}^{0}\right)-\kappa_{0}/2\right]p_{-}-i\mu \mu_{0}E_{s}w_{-},$$

(30) $$0=-\kappa_{0}\mu \left(w_{0}+1\right)+i2\left(\mu_{0}{E_{s}p}_{0}^{*}-\mu_{0}^{*}{E_{s}^{*}p}_{0}\right),$$

(31) $$i\delta \mu w_{-}=-\kappa_{0}\mu w_{-}+i2\left(\mu_{0}{E_{s}p}_{+}^{*}-\mu_{0}^{*}{E_{s}^{*}p}_{-}-\mu_{0}{E_{w}p}_{0}^{*}\right).$$

After the mathematical calculations, we can obtain the solution for $p_{+}$ as

(32) $$p_{+}=\frac{\mu_{0}\mu E_{w}H\left(\delta \right)w_{0}}{D(\delta )},$$

where,

(33) $$D\left(\delta \right)=4|\mu_{0}{E_{s}|}^{2}\left(\delta +i\kappa_{0}/2\right)+\left(\delta +i\kappa_{0}\right)\left[\left(\omega -\omega_{ex}^{0}\right)-\delta -i\kappa_{0}/2\right][\left(\omega -\omega_{ex}^{0}\right)+\delta +i\kappa_{0}/2],$$

(34) $$H\left(\delta \right)=\left(\delta +i\kappa_{0}\right)\left[\left(\omega -\omega_{ex}^{0}\right)-\delta -i\kappa_{0}/2\right]+\frac{2|\mu_{0}{E_{s}|}^{2}\delta }{\left(\omega -\omega_{ex}^{0}\right)-i\kappa_{0}/2},$$

(35) $$w_{0}=-\frac{{\left(\omega -\omega_{ex}^{0}\right)}^{2}+\kappa_{0}^{2}/4}{{\left(\omega -\omega_{ex}^{0}\right)}^{2}+\kappa_{0}^{2}/4+2|\mu_{0}{E_{s}|}^{2}}.$$

Here, $\rho_{22}^{s}=\left(w_{0}+1\right)/2$ and $\rho_{22}^{w}=2Im\left[\mu_{0}^{*}E_{w}^{*}p_{+}\right]/\left(\mu \kappa_{0}\right)$ represent the contributions of strong field and weak field to the exciton population, respectively. It is obvious, $\rho_{22}^{s}\gg \rho_{22}^{w}$. Using Equation (32), the energy absorption rate of the SQD for the weak laser field can be expressed as $Q_{S}^{w}\left(\delta \right)=\hbar \omega_{w}\kappa Im[K]$, where

(36) $$K=\frac{{|\mu_{0}E_{w}|}^{2}w_{0}}{D\left(\delta \right)\kappa_{0}}\times \left\{\left(\delta +i\kappa_{0}\right)\left[\left(\omega_{s}-\omega_{ex}^{0}\right)-\delta -i\kappa_{0}/2\right]+\frac{2|\mu_{0}{E_{s}|}^{2}\delta }{\left[\left(\omega_{s}-\omega_{ex}^{0}\right)-i\kappa_{0}/2\right]}\right\}.$$

However, the energy absorption rate of the MNP is given by

(37) $$Q_{M}^{w}\left(\delta \right)=\frac{\left(\omega_{s}+\delta \right){\left|E_{w}\right|}^{2}R^{3}Im\left[\varepsilon_{M}\left(\omega_{s}+\delta \right)\right]}{6|\varepsilon_{eff1}{|}^{2}}\times \frac{|\omega_{ex}^{0}-\omega_{s}-\delta +\theta \mu_{R}{|}^{2}+{\left[\theta \mu_{I}-\kappa_{0}/2\right]}^{2}+2{\left|\mu_{0}E_{w}\right|}^{2}}{{\left[\omega_{ex}^{0}-\omega_{s}-\delta \right]}^{2}+\kappa_{0}^{2}/4+2{\left|\mu_{0}E_{w}\right|}^{2}}.$$

**Figure 4:**
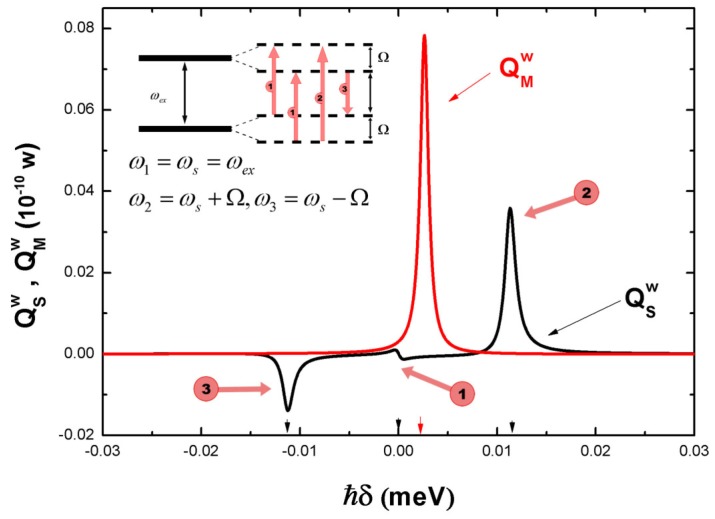
The energy absorption rate of the SQD ($Q_{S}^{w}\left(\delta \right)$) and the MNP ($Q_{M}^{w}\left(\delta \right)$) to the weaklaser field. Inset: Quantum transitions of SQD’ subsystem corresponding to the three absorption peaks.

Figure 4 shows the energy absorption rate of the SQD ($Q_{S}^{w}\left(\delta \right)$), the MNP ($Q_{M}^{w}\left(\delta \right)$) to the weak laser field when $\omega_{s}=\omega_{ex}$, $\varepsilon_{0}=\varepsilon_{S}=12$ and d=30 nm. Other parameters are the same as the above section. In solid state system, controlling light with light helps to implement various optical processing devices. In the hybrid SQD-MNP system, in principle we illustrate that the energy absorption rate of the system to the weak laser field can be tuned by the strong laser field. Here, we focus on the response of the system to the weak laser field. In Figure 4, the sharp peak in the middle (the red curve) represents the energy absorption rate of the MNP to the weak laser field. The other three peaks (the black curve) corresponding to three quantum transitions as shown in inset illustrate the energy absorption rate of the SQD to the weak laser field [40]. Their corresponding transition frequency can be tuned by the strong laser intensity. For instance, the frequency $\omega_{3}=\omega_{s}+\Omega$ (corresponding to the second peak with number 2), where $\Omega =\sqrt{{\left(\omega_{s}-\omega_{ex}^{0}\right)}^{2}+4(|\mu_{0}{E_{s}\left|/\hbar \right)}^{2}}$ depends strongly on $|{E_{s}|}^{2}$. So, it is possible to control the optical absorption to weak laser field with strong laser field in the coupled SQD-MNP system. The optical property of the coupled SQD-MNP system may be applied in the optical processing device in the future.

The above discussion is under the quantum description for the exciton-plasmon interaction. In what follows, we compare the quantum description with the semiclassical description. In Ref. [41], Lu and Zhu investigated the hybrid SQD-MNP system in the presence of a strong pump field and a weak probe field under the semiclassical description, and revealed slow light effect appeared in this system. However, electromagnetically induced transparency (EIT) is often exploited to implement slow light [42].

**Figure 5:**
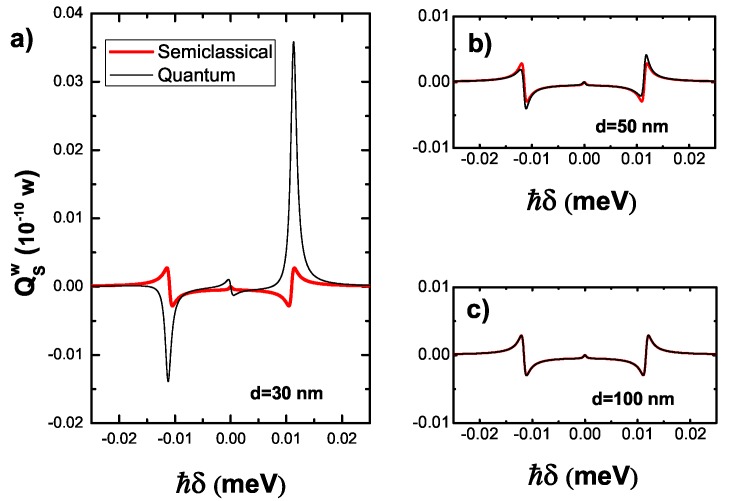
The energy absorption rate of the SQD under the both quantum and semiclassical descriptions for different distances d= 30 (**a**),50 (**b**),100 (**c**) nm.

Here, we study the energy absorption rate of the SQD to the weak laser field under both semiclassical and quantum descriptions with the same parameters in Figire Figure 4. In Figure 5, the red thick curves (the black thin curves) represent the results of the semiclassical (quantum) description for different distances d=30,50,100 nm. When d=30 nm, the result of quantum description deviate significantly from that of semiclassical description. However, the deviation becomes slight with the increasing distance. In Figure 5c the results of the two descriptions are identical when d=100 nm. We note that the increase of the distance produces little difference to the result of the semiclassical description. In contrast, the result of the quantum description has a huge change, especially for the short distance. The rapid change is caused by the strong exction-plasmon interaction. Quantum description can pave the way for the investigation of the optical properties induced by the strong exciton-plasmon interaction.

## 4. Quantum Entanglement of Two SQDs Induced by a MNP

Recently, the coupling among SQDs mediated by surface plasmon field has received increasing attention [22,43]. Here, we consider two SQDs in the vicinity of a MNP. Each SQD consists of the electronic ground state $|0\rangle$ and the first excited state $|ex\rangle$. They interact with surface plasmon field in the MNP. Firstly, we need to quantize surface plasmon field based on the cavity quantum electrodynamics. Recently, a good deal of work had been devoted to quantize surface plasmon field in the metal [14,18,19,44,45]. surface plasmon field in the MNP can be considered as a multi-modes field. After the second quantization of surface plasmon field, the Hamiltonian can be written as $H_{P}=\sum_{k}\omega_{k}a_{k}^{+}a_{k}$ [18,19], where $\omega_{k}$ is the frequency of surface plasmon field mode *k*, $a_{k}^{+}$($a_{k}$) is the creation (annihilation) operator of surface plasmon field mode *k*. Next, we consider the interaction between each SQD and surface plasmon field modes. We assume that the coupling strength between each SQD and surface plasmon field is identical for simplicity. The interaction Hamiltonian, under the rotating-wave approximation, can be written as $H_{int}=-\sum_{k}{(g_{k}a_{k}\sigma }_{+}+g_{k}^{*}a_{k}^{+}\sigma )$ [14,30], where $g_{k}$ is the coupling strength between each SQD and surface plasmon field mode *k*, $\sigma_{+}=\sigma_{+}^{1}+\sigma_{+}^{2},\sigma_{+}^{i}={|ex\rangle }_{i}\langle 0|$ is the raising operator of the *i*th SQD. Therefore, the Hamiltonian of the entire system can be written as (ℏ=1)

(38) $$H=\omega_{ex}(\sigma_{z}^{1}+\sigma_{z}^{2})+\sum_{k}[\omega_{k}a_{k}^{+}a_{k}-{(g_{k}a_{k}\sigma }_{+}+g_{k}^{*}a_{k}^{+}\sigma )],$$

where $\sigma_{z}^{i}=\left(1/2\right)\times \left({|ex\rangle }_{i}\langle ex|-{|0\rangle }_{i}\langle 0|\right).$ The full quantum dynamics of the coupled nanosystem can be derived from the following master equation for the density operator $\partial_{t}\rho =-i\left[H,\rho \right]+\varsigma_{SQD}+\varsigma_{SPP}$, with the Liouvillian terms [32], $\varsigma_{SQD}=\left(\kappa /2\right)\times \sum_{i=1,2}\left(2\sigma_{-}^{i}\rho \sigma_{+}^{i}-\rho \sigma_{+}^{i}\sigma_{-}^{i}-\sigma_{+}^{i}\sigma_{-}^{i}\rho \right)$ describes the decay of each SQD to Markovian reservoirs, κ is the exciton radiative decay rate in SQDs, $\varsigma_{SPP}=\sum_{i}\left(\gamma_{k}/2\right)\times \left(2a_{k}\rho a_{k}^{+}-\rho a_{k}^{+}a_{k}-a_{k}^{+}a_{k}\rho \right)$ describes the relaxation of surface plasmon field mode *k* with decay rate $\gamma_{k}$. Next, we take a time-independent unity transformation $e^{is}$ on the density operator, where $s=\sum_{i,k}{(\pi_{k}a_{k}\sigma }_{+}^{i}{+\pi_{k}^{*}a_{k}^{+}\sigma }^{i})$, $\pi_{k}=2g_{k}/(\gamma_{k}+2i\delta_{k})$, $\delta_{k}=\omega_{k}-\omega_{ex}$, so that $\tilde{\rho }=e^{is}\rho e^{-is}$. If $\left|\pi_{k}\right|\ll 1$, the second-order term remains, and the higher-order terms can be ignored safely. Thus, for the reduce density operator of SQDs, we have $\partial_{t}\rho_{SQD}=-i\left[H_{eff},\rho_{SQD}\right]+\varsigma_{SQD}^{\prime }$, where $\rho_{SQD}=Tr_{P}\left[\tilde{\rho }\right]$. The effective Hamiltonian to reveal the exciton energy shift and the coupling among SQDs is given by

(39) $$H_{eff}={(\omega_{ex}-\eta }_{0})(\sigma_{z}^{1}+\sigma_{z}^{2})-\eta \left(\sigma_{+}^{1}\sigma_{-}^{2}+\sigma_{-}^{1}\sigma_{+}^{2}\right),$$

where $\eta_{0}=\eta +\sum_{k}8{\left|g_{k}\right|}^{2}\delta_{k}{\bar{n}}_{k}/\left(\gamma_{k}^{2}+4\delta_{k}^{2}\right)$, ${\bar{n}}_{k}=<a_{k}^{+}a_{k}>$, $\eta =\sum_{k}4{\left|g_{k}\right|}^{2}\delta_{k}/\left(4\delta_{k}^{2}+\gamma_{k}^{2}\right)$ is the coupling strength among SQDs induced by surface plasmon field modes. At low temperature, ${\bar{n}}_{k}\ll 1$, so that $\eta_{0}≅\eta$. The dissipation term is given by

(40) $$\varsigma_{SQD}^{{}^{\prime }}=(\Gamma_{i,j}/2)\times \sum_{i,j}(2\sigma_{-}^{i}\rho_{SQD}\sigma_{+}^{j}-\sigma_{+}^{i}\sigma_{-}^{j}\rho_{SQD}-\rho_{SQD}\sigma_{+}^{i}\sigma_{-}^{j}),$$

$\Gamma_{i,j}=\kappa +2\tau$ if i=j, $\Gamma_{i,j}=2\tau$ if i≠j, where $\tau =\sum_{k}2{\left|g_{k}\right|}^{2}\gamma_{k}/\left(4\delta_{k}^{2}+\gamma_{k}^{2}\right)$. We note that a cross-decay rate 2τ between the two SQDs appears and the exciton lifetime decreases because of the presence of surface plasmon field field. The cross-decay rate represents the nonradiative decay rate that can be decomposed into different contributions for each surface plasmon field mode, i.e., $2\tau ≅\Gamma_{MNP}^{nr}$ [14]. According to the quantum-semiclassical correspondence, we have $\eta =Re[G\left(\omega_{ex}\right)],$ $\tau =Im[G\left(\omega_{ex}\right)]$.

Our method to treat the Hamiltonian is similar with Schrieffer-Wolff transformation which can be used in cavity (circuit) QED system [46]. Circuit QED system has been received extensive attention in recent years. Superconducting circuits in circuit QED system exhibit macroscopic quantum coherence. So, it can behave like artificial atoms, which can be investigated by cavity QED theory. You et al. have presented a brief overview of the latest progress in this rapidly advancing field [47,48]. Superconducting circuits can interact with other quantum systems such as atom, spin etc., in order to implement some hybrid circuits [49]. Hybrid circuit fabricated on a chip is crucial for building future quantum technologies, including quantum simulators [50,51], and quantum computers [52]. In cavity (circuit) QED, when the decay rate of cavity mode is very small as compared to the detuning between the cavity mode frequency and the transition frequency of qubits so that it can be ignored safely, the effective Hamiltonian can be obtained by using Schrieffer-Wolff transformation [53,54]. Under the treatment of Schrieffer-Wolf transformation, one can obtain $\eta =\sum_{k}{\left|g_{k}\right|}^{2}/\delta_{k}$, τ=0. But it is well-known that the decay of surface plasmon field is too large to be ignored in the coupled SQD-MNP system. Taking this fact fully into account, our method is suitable for revealing the exciton energy shift, the modify decay rate and the coupling strength among SQDs.

When the distances between every SQD and the MNP are not equal ($d_{1}\neq d_{2}$), we need to make a modification for the expression of two parameters η, τ. If one of the two distances changed, the expressions of the cross-decay rate and the coupling constant between the two SQDs need to be modified. As mentioned above, $g_{k}∼d^{-3}$. The expression of the cross-decay rate and the coupling strength can be rewritten as $Im[G\left(\omega_{ex}\right)]$ and $Re[G\left(\omega_{ex}\right)]$, respectively, where $G\left(\omega_{ex}\right)=\left[\gamma {\left(\mu s_{\alpha }\right)}^{2}R^{3}]/[\hbar \varepsilon_{0}\varepsilon_{eff1}^{2}d_{1}^{3}d_{2}^{3}\right]$. However, here, we assume that $d_{1}=d_{2}=d$ for simplicity. In the SQDs’ subsystem, we choose an adequate basic of SQDs’ subsystem, i.e., $|1\rangle =|0,0\rangle$, $|2\rangle =\left(1/\sqrt{2}\right)\times \left(|ex,0\rangle +|0,ex\rangle \right)$, $|3\rangle =\left(1/\sqrt{2}\right)\times \left(|ex,0\rangle -|0,ex\rangle \right)$, $|4\rangle =|ex,ex\rangle$. The four collective states are the eigenstates of the two coupling SQDs. The master equation of the SQDs’ subsystem is given by

(41) $$\partial_{t}\rho =-i\left[H^{\prime \prime },\rho \right]+\zeta_{SQD},$$

where $H^{\prime \prime }=-\left(\omega_{ex}-\eta \right)|1\rangle \langle 1|-\eta |2\rangle \langle 2|+\eta |3\rangle \langle 3|+\left(\omega_{ex}-\eta \right)|4\rangle \langle 4|$, $\zeta_{SQD}\left(\rho \right)=\left[\left(\kappa +4\tau \right)/2\right]\times \left[2\left(|2\rangle \langle 4|+|1\rangle \langle 2|\right)\rho \left(|4\rangle \langle 2|+|2\rangle \langle 1|\right)-\left(|2\rangle \langle 2|+|4\rangle \langle 4|\right)\rho -\rho \left(|2\rangle \langle 2|+|4\rangle \langle 4|\right)\right]+\left(\kappa /2\right)\times \left[2\left(|1\rangle \langle 3|-|3\rangle \langle 4|\right)\rho \left(|3\rangle \langle 1|-|4\rangle \langle 3|\right)-\left(|3\rangle \langle 3|+|4\rangle \langle 4|\right)\rho -\rho \left(|3\rangle \langle 3|+|4\rangle \langle 4|\right)\right].$ It shows two dissipated channels. The first term describes dissipation through one cascade channel $|4\rangle \to |2\rangle \to |1\rangle$ with fast decay rate κ+4τ. The second term describes dissipation through another cascade channel $|4\rangle \to |3\rangle \to |1\rangle$ with slow decay rate κ.

In order to illustrate the coupling of the two SQDs, we analyze the following two parameters: (1) The probability of the two SQDs being in the state $|i\rangle$, $P_{i}\left(t\right)=\rho_{i,i}\left(t\right)$, for i=1,2,3,4. (2) The concurrence for quantifying entanglement of the two SQDs, $C\left(t\right)=\sqrt{{\left[\rho_{2,2}\left(t\right)-\rho_{3,3}\left(t\right)\right]}^{2}+4Im{\left[\rho_{2,3}\left(t\right)\right]}^{2}}$ [22,55]. Here we use the above parameters, and take d=18 nm.

**Figure 6:**
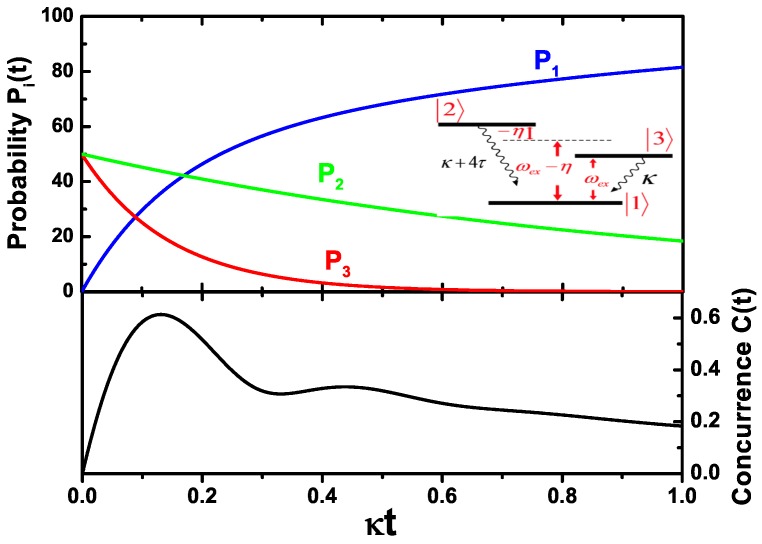
The probability of each state, the concurrence of the two SQDs as a function of time when the initial state of the two SQDs is the state $|ex,0\rangle$. The inset shows the dissipation channels of the two SQDs.

If the initial state of the two SQDs is prepared in a product state $|ex,0\rangle$, only two dissipation channels $|2\rangle \to |1\rangle$ and $|3\rangle \to |1\rangle$ should been considered (see inset of Figure 6). As shown in Figure 6, with the decrease of $P_{2}\left(t\right)$ and $P_{3}\left(t\right)$, the probability of two SQDs in the state $|1\rangle$ increases. At about $t=0.14\kappa^{-1}$, the concurrence of two SQDs reaches the maximal value. In the figure of the concurrence, a weak oscillation is presented as a result of the coupling of the two SQDs.

**Figure 7:**
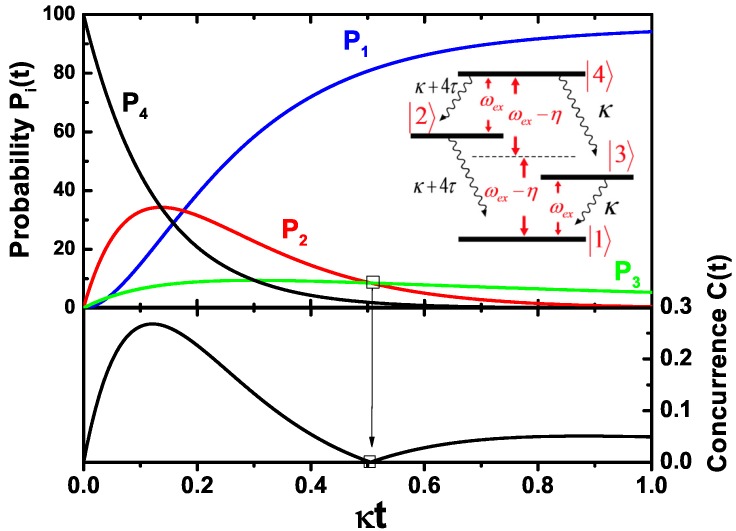
The probability of each state, the concurrence of the two SQDs as a function of time when the initial state of every SQD is in their excited state. The inset shows the dissipation channels of the two SQDs.

Another case is that the initial state is in the state $|ex,ex\rangle$. Figure 7 shows the probability of each state, the concurrence as a function of time. It is surprising that the two SQDs being in their excited states can be entangled for a long time. Only at about $t_{0}=0.51\kappa^{-1}$ the concurrence is equal to zero (see the figure of the concurrence); and $P_{2}\left(t_{0}\right)=P_{3}\left(t_{0}\right)$ (see the figure of probability). This is because two entangled states $|2\rangle$ and $|3\rangle$ make a product state $|ex,0\rangle$ or $|0,ex\rangle$. The absence of the oscillation in the figure of the concurrence implies that the coupling of the two SQDs can not play a role in the creation of the concurrence. Moreover, a stationary state with a high concurrence can be achieved by continuous pumping [22]. Our results illustrated that the plasmon field of a MNP can lead to the entanglement of two SQDs. Furthermore, an array of metal nanoparticles can be used to generate the entanglement of two SQDs, which has been reported by Lee et al. [56]. The entanglement generated between two SQDs is because of the energy transfer via the array of metal nanoparticles. Interestingly, an array of cavities can be considered as a quantum way to transfer energy, which can be exploited to implement single-photon transport [39,57,58,59].

**Figure 8:**
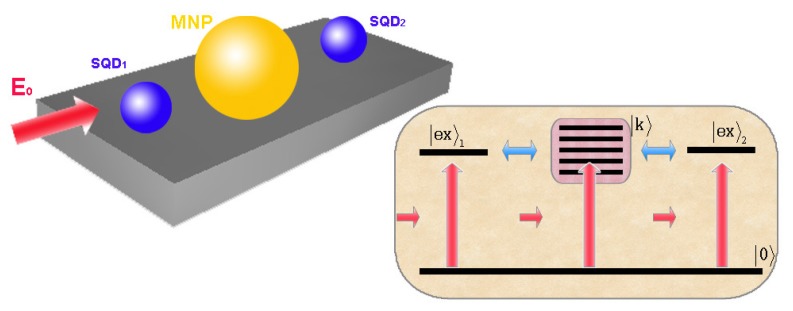
Schematic illustration of a hybrid molecule consisting of two identical SQDs (SQD 1 and SQD 2) and a MNP in the presence of the pump laser field $E_{0}$. Inset shows quantum transitions (including photon-induced transition and coupling-induced transition) in the hybrid molecule.

In this hybrid system, a pump laser field $E_{0}=Ee^{-i\omega t}+c.c.$ can be used to excite the two SQD, as illustrated in Figure 8.

The total Hamiltonian can be written as

(42) $$H=\hbar \omega_{ex}\sigma_{z}+\hbar \sum_{k}\left(\omega_{k}a_{k}^{+}a_{k}\right)-\hbar \sum_{k}\left(g_{k}a_{k}\sigma_{+}+g_{k}^{*}a_{k}^{+}\sigma \right)-\left\{\left[\left(\mu /\varepsilon_{eff}\right)\sigma_{+}+\sum_{k}\left(\mu_{k}^{*}a_{k}^{+}\right)\right]Ee^{-i\omega t}+h.c.\right\},$$

where $\sigma_{z}=\sigma_{z}^{1}+\sigma_{z}^{2},$ μ ($\mu_{k}$) is the dipole moment between the ground state and the excited exciton state ${|ex\rangle }_{i}$ of SQD (the excited plasmon state |k〉 of MNP), $\varepsilon_{eff}=\left(\varepsilon_{s}+2\varepsilon_{0}\right)/3\varepsilon_{0}$ is the screening factor with $\varepsilon_{s}$ being the dielectric constant of SQD.

Based on the master equation of this hybrid system, the expectation values $A_{k}$, *B* and $\langle a_{k}^{+}a_{k}\rangle$ satisfy the following equations

(43) $$\hbar \partial_{t}A_{k}=-i\hbar \left(\omega_{k}-\omega -i\gamma_{k}/2\right)A_{k}+i\left(\hbar g_{k}^{*}B+\mu_{k}^{*}E\right),$$

(44) $$\hbar \partial_{t}\langle a_{k}^{+}a_{k}\rangle =-\hbar \gamma_{k}\langle a_{k}^{+}a_{k}\rangle -i\left[A_{k}\left(\hbar g_{k}B^{*}+\mu_{k}^{*}E\right)-c.c.\right],$$

where $A_{k}=\langle a_{k}e^{i\omega t}\rangle$, $B=\langle \sigma e^{i\omega t}\rangle .$ The above equations can not illustrate the dynamics of the entire system, but show the correspondence among some expectation values. In the steady state limit, making use of the above equations we have $A_{k}=\left(\hbar g_{k}^{*}B+\mu_{k}^{*}E\right)/\hbar \left(\omega_{k}-\omega -i\gamma_{k}/2\right)$.

We now can take a time-independent unity transformation on the orignal master equation. The master equation of the SQDs’ subsystem can be written as (ℏ=1)

(45) $$\partial_{t}\rho_{S}=-i\left[H_{S},\rho_{S}\right]+\varsigma_{S}$$

where

(46) $$H_{S}=\omega_{0}\sigma_{z}+G_{0}\left(\sigma_{+}^{1}\sigma^{2}+\sigma^{1}\sigma_{+}^{2}\right)-\left(\mu_{0}E\sigma_{+}e^{-i\omega t}+c.c.\right),$$

(47) $$\varsigma_{S}=\sum_{i,j=1,2}\left(\Gamma_{i,j}/2\right)\left(2\sigma^{j}\rho_{S}\sigma_{+}^{i}-\sigma_{+}^{i}\sigma^{j}\rho_{S}-\rho_{S}\sigma_{+}^{i}\sigma^{j}\right),$$

$\omega_{0}=\omega_{ex}-G_{R}$, $\mu_{0}=\mu /\varepsilon_{eff}+C\left(\omega_{ex}\right)$, $\Gamma_{1,1}=\Gamma_{2,2}=\kappa_{0}$, $\Gamma_{1,2}=\Gamma_{2,1}=\kappa_{1}$, $G_{R}=Re\left[G\left(\omega_{ex}\right)\right]$, $G_{I}=Im\left[G\left(\omega_{ex}\right)\right]$, $\kappa_{0}=\kappa +2G_{I}$, $\kappa_{1}=2G_{I}$. The above master equation illustrates the coupling of two SQDs (the coupling constant $G_{0}=-G_{R}$). The quantized plasmon field produced in the MNP plays the platform of Förster energy transfer between two SQDs [60]. The master equation derived by the quantum transformation method is in good agreement with that of Ref. [22] which describes the interaction between two qubits mediated by one-dimensional plasmon field. In the SQDs’ subsystem, we choose an adequate basic, i.e., |1〉=|0,0〉, $|2\rangle =(|0,ex\rangle +\left|ex,0\rangle \right)/\sqrt{2}$, $|3\rangle =(|0,ex\rangle -\left|ex,0\rangle \right)/\sqrt{2}$, |4〉=|ex,ex〉. Based on Equations (43) and (44), the exciton population *M* satisfies the following equations

(48) $$\partial_{t}B=-i\left\{\left[\omega_{0}-\omega -i\left(\kappa_{0}+\kappa_{1}\right)/2\right]+G_{R}B\left(M-1\right)-2\mu_{0}E\left(M-1\right)\right\}$$

(49) $$\partial_{t}M=-\left(\kappa_{0}+\kappa_{1}\right)M-i\left(\mu_{0}EB^{*}-c.c.\right)$$

Based on the above equations, we can obtain the steady-state solution $B=2\Omega_{0}\left(M-1\right)/\left(K-i\right)$, where $K=2\left[\left(\omega_{ex}-2G_{R}-\omega \right)+MG_{R}\right]/\left(\kappa_{0}+\kappa_{1}\right)$, $\Omega_{0}=2\mu_{0}E/\left(\kappa_{0}+\kappa_{1}\right)$. The exciton population *M* is determined by the following equation

(50) $$\left(K^{2}+1\right)M+2{\left|\Omega_{0}\right|}^{2}\left(M-1\right)=0$$

We exploit a quantum transformation to reduce the direct coupling between SQDs and MNP, so that their coupling are mainly included in the terms of high order which can be neglected for obtaining the master equation of SQDs’ subsystem. This is a transformational decoupling treatment. After the treatment, however, the SQDs’ subsystem is not considered as a closed system because the obtained steady-state solutions, such as *M*, have included “information” of the plasmon modes of MNP.

The energy transfer between SQDs and MNP occurs in the hybrid molecule [5,61,62]. The exciton energy can be transferred from SQDs to MNP, and then converted into heat [3,6]. The energy absorption rate of MNP, $Q_{M}=\sum_{k}\hbar \omega_{k}\gamma_{k}\langle a_{k}^{+}a_{k}\rangle /2$, can be obtained by Equation (45). In the steady-state limit, Combining Equations (46) and (47) we have

(51) $$\langle a_{k}^{+}a_{k}\rangle =\frac{|g_{k}{B|}^{2}+{\left|\mu_{k}E\right|}^{2}/\hbar^{2}+2Re\left[g_{k}^{*}\mu_{k}B\right]}{{\left(\omega_{k}-\omega \right)}^{2}+\gamma_{k}^{2}/4}.$$

Since $g_{k}=\theta \mu_{k}$, ($\theta =\mu s_{\alpha }/\left(\hbar \varepsilon_{0}\varepsilon_{eff}d^{3}\right)$ is independent on the index k), we can obtain

(52) $$Q_{M}=Q_{0}\times \frac{{\left(K-q_{R}\right)}^{2}+{\left(q_{I}+1\right)}^{2}}{K^{2}+1},$$

where $Q_{0}=\sum_{k}\omega_{k}\gamma_{k}{\left|\mu_{k}\right|}^{2}E^{2}/\left\{2\hbar \left[{\left(\omega_{k}-\omega \right)}^{2}+\gamma_{k}^{2}/4\right]\right\}$, $q_{R}=Re\left[q\right]$, $q_{I}=Im\left[q\right]$, $q=4\theta \left(1-M\right)\mu_{0}/\left(\kappa_{0}+\kappa_{1}\right)$. Based on Equation (12), $Q_{0}=Im\left[\omega G\left(\omega \right)/\theta^{2}\right]=\omega \varepsilon_{0}Im\left[\gamma \right]R^{3}E^{2}/2$ [15]. Equation (52) shows a Fano function [23] with the generalized field–dependent complex Fano factor which includes both the nonlinear and dephasing effects [15]. However, the energy absorption rate of SQDs’ subsystem is given by $Q_{S}=\hbar \omega_{ex}\kappa M/2$. As an example, we consider a Au MNP with radius R=7 nm, and its dielectric constant is ${\varepsilon_{M}\left(\omega \right)=\varepsilon }_{b}-\omega_{p}^{2}/\left[\omega \left(\omega +i\eta \right)\right]$ with $\varepsilon_{b}=9.5$, $\hbar \omega_{p}=9$ eV, ℏη=0.07 eV [36]. The dielectric constant of the background medium is $\varepsilon_{0}=2$ (polymer), and $\varepsilon_{s}=7.2$ (CdTe). And $s_{\alpha }=2$. For the decay rate and the dipole moment of the exciton, we take κ=2.5 GHz, and $\mu =er_{0}$ with $r_{0}=0.65$ nm.

**Figure 9:**
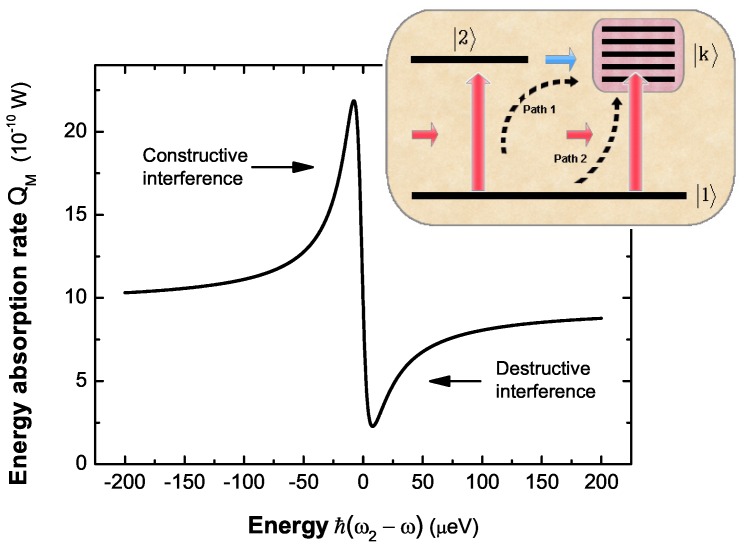
The energy absorption rate of the MNP as a function of the energy difference $\hbar (\omega_{2}-\omega_{ex})$ with the pump laser intensity $I_{0}=800$ W/cm${}^{2}$. Inset: Quantum interference pathways to excite the plasmon states |k〉 in the hybrid molecule in the steady-state limit.

Figure 9 shows the nonlinear Fano profile in the energy absorption spectrum of MNP in the strong pump laser field ($I_{0}=800$ W/cm${}^{2}$). The Fano interference refers to three states [24], i.e., the common ground state |1〉, an entangled state |2〉 and an infinite collection of extended states |k〉. As shown in inset of Figure 9, there are two optical excitation transitions (|1〉→|2〉 and |1〉→|k〉) and one coupling transition (|2〉→|k〉). Two optical pathways (path 1 and 2 in inset) can be found to generate the continuum of states |k〉. Constructive or destructive interference between two pathways, depending on the energy difference between quantum transition ($\hbar \omega_{2}\equiv \hbar \left(\omega_{ex}-2G_{R}\right)$ represents the quantum transition energy between |1〉 and |2〉) and the pump laser (ℏω), give rise to the nonlinear Fano effect (see Fano function in Equation (52)).

**Figure 10:**
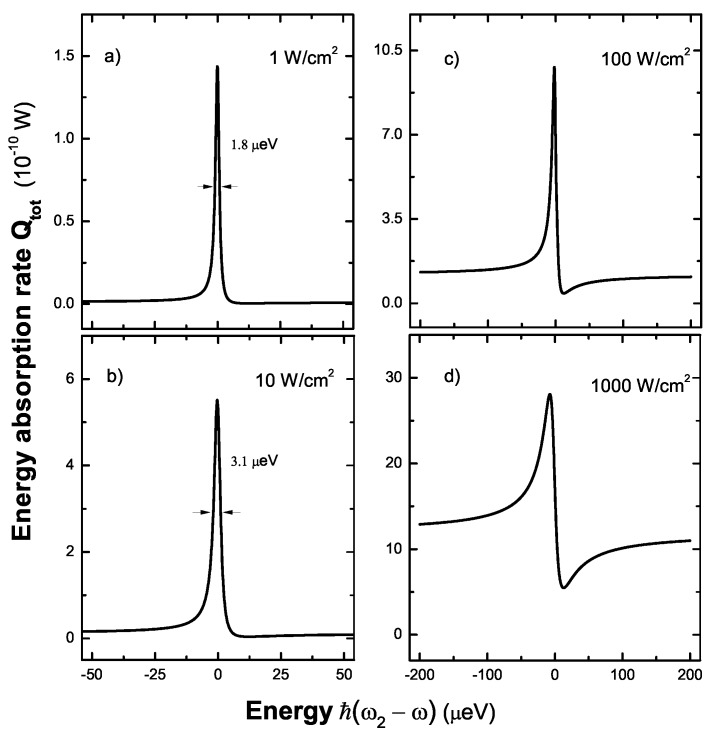
The total energy absorption rate as a function of the energy difference $\hbar (\omega_{2}-\omega_{ex})$ with different pump laser intensity $I_{0}$, (**a**) 1 W/cm${}^{2}$; (**b**) 10 W/cm${}^{2}$; (**c**) 100 W/cm${}^{2}$; (**d**) 1000 W/cm${}^{2}$.

The total energy absorption rate of the entire system is given by $Q_{tot}=Q_{M}+Q_{S}$. In the weak field regime, the linear Fano effect appears in the total energy absorption spectrum. Figure 10a,b shows a symmetric peak with the broadening 1.8 μeV (3.1 μeV) as the pump laser intensity $I_{0}$=1 W/cm${}^{2}$ (10 W/cm${}^{2}$). The exciton population M≪1 in the presence of weak pump laser field so that the Fano factor $q_{R}\gg 1$, which lead to the appearance of the symmetric peak profile [23]. However, the asymmetric Fano profile becomes more and more pronounced as the pump laser intensity increases. As shown in Figure 10c,d, we can see the obvious nonlinear Fano effect as a result of the Fano interference. A strong pump laser field creates a large exciton population in SQDs’ subsystem which give rise to the appearance of the nonlinear Fano effect. We find that the exciton population, which depends on the pump laser intensity, is a important factor in the determination of Fano profile. The entangled state |2〉 is a state in one of the two optical pathways that create Fano effect, which gives us a motivation to connect Fano profile with entanglement of two SQDs. Concurrence for quantifying entanglement of two SQDs, can be expressed as $C\left(t\right)=\sqrt{{\left[\rho_{2,2}\left(t\right)-\rho_{3,3}\left(t\right)\right]}^{2}+4Im{\left[\rho_{2,3}\left(t\right)\right]}^{2}}$ [22,55].

**Figure 11:**
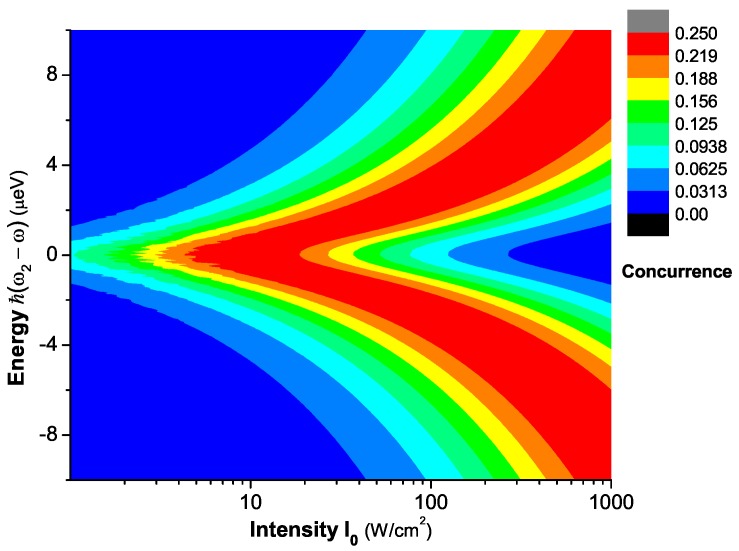
The steady-state concurrence versus the energy difference $\hbar (\omega_{2}-\omega )$ and the pump laser intensity $I_{0}$.

**Figure 12:**
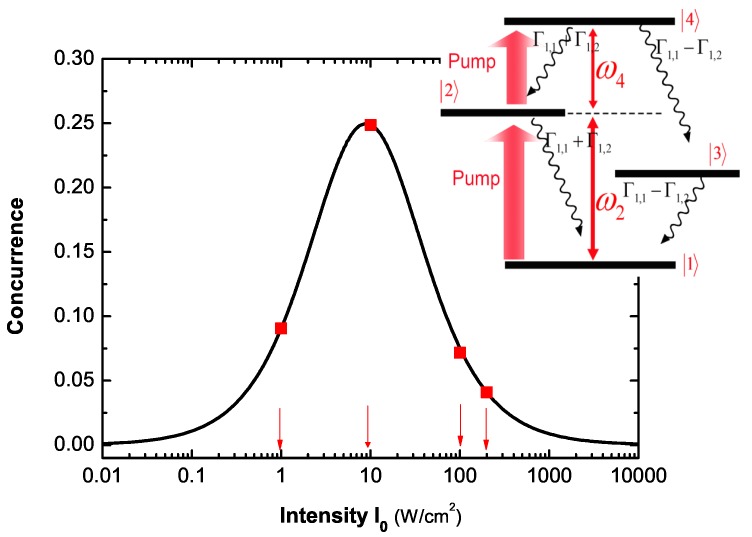
The steady-state concurrence as a function of the pump intensity $I_{0}$ at resonance $\omega_{2}=\omega$. Inset: The energy levels of two coupled SQDs.

Figure 11 plots the steady-state concurrence versus the energy difference $\hbar (\omega_{2}-\omega )$ and the pump laser intensity $I_{0}$. We see that the steady-state concurrence at resonance reaches the maximum value for every fixed intensity. The pump laser intensity and the energy difference can be obtained by analyzing Fano profile based on Figure 10. Combining the two important parameters with Figure 11, then, we can evaluate the steady-state concurrence. Because the entanglement of two SQDs is determined by both the pump intensity and the energy difference, according to the red region of Figure 11, we can properly choose the two parameters to obtain the non-negligible entanglement. We can also see that the steady-state concurrence at resonance reaches the maximum value for every fixed intensity. At resonance $\omega_{2}=\omega$, we plot the steady-state concurrence as a function of the pump intensity $I_{0}$. If the pump rate is much slower than the life of the state |2〉 (corresponding to a very weak pump intensity, such as 0.01 W/cm${}^{2}$), the state |2〉 can be hardly populated so that the populations of the states |2〉, |3〉 and |4〉 can be neglected (the population of the state |4〉 is not more than that of the state |2〉 because of pumping from |2〉 to |4〉). This is the reason why the steady-state concurrence approximates to zero in the presence of very weak pump intensity. Because the transition frequency $\omega_{2}$ from |1〉 to |2〉 is equal to the pump laser frequency ω (resonance, see the inset of Figure 12), the population of the state |2〉 can reach the saturation with an appropriate pump intensity (whose pump rate is much faster than the life of the state |2〉). Compared to the former, a stronger pump intensity is needed for the population of the state |4〉 to reach the saturation because of the non-resonance (see the inset of Figure 12) between the pump frequency ω and the transition frequency $\omega_{4}$ from |2〉 to |4〉 ($\omega_{4}=\omega_{ex}$). And the population of the state |4〉 is connected to that of the state |3〉. With the increasing pump intensity, therefore, the steady-state concurrence firstly increases to reach the maximum value, then decreases. When the pump intensity is very large, such as 10000 W/cm${}^{2}$, the populations of the three states, |2〉, |3〉 and |4〉 reach the saturation, and they are approximately equal so that the steady-state concurrence also approximates to zero. From Figure 12, the steady-state concurrence reaches its maximum value at an optimal pump intensity, about 10 W/cm${}^{2}$. However, the steady-state concurrence is small at strong pump intensity, such as 100 W/cm${}^{2}$, 200 W/cm${}^{2}$. So, at resonance we can choose an appropriate pump intensity region for detecting the non-negligible entanglement.

Entanglement exists in various physical systems, such as entanglement based on photons [63], atoms [64], SQDs [43] and polymer molecules [65]. Quantum state tomography can be extensively used to measure the entangled state [66]. However, it is challenging in experiment because many copies of the measured states are necessary. The optical observation proposed by us, here, is a simple and feasible approach to obtain information of entanglement. And entanglement remains after the observation. The novel approach has potential to reveal entanglement in many solid-state systems. In Ref. [67], the authors demonstrated the generation of entanglement between two distant qubits mediated by the plasmonic waveguide, and proposed a scheme to detect the entanglement by measuring the cross terms of a second-order coherence function. The correlations between entanglement and Fano resonance have been reported by Chen et al. [68]. They investigated the Fano resonance of the scattering spectra in a system consisting of a metal nanowire coupled to two colloidal quantum dots, and revealed that there exists correlations between the entanglement of the two QDs and the Fano resonance. In Ref. [69], Buscemi et al. proposed that the production and detection of carrier-carrier entanglement in quantum dot structures may be controlled by the manipulation of Fano resonances in the transmission spectra.

## 5. Fano Correlation Effect of Two MNPs Coupling to a SQD

**Figure 13:**
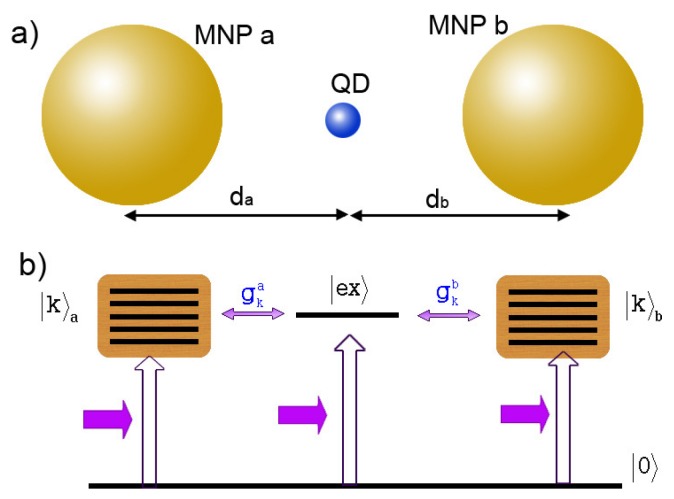
(**a**) A QD is placed in the gap of two identical MNPs (MNP a and b) with radius *R* in the presence of an external field, and their center-to-center distances are $d_{a}$ and $d_{b}$; (**b**) Quantum transitions (including photon-induce transition and coupling-induce transition) in the hybrid molecule.

We consider a hybrid molecule consisting of two identical spherical MNP (MNP a and MNP b) with radius *R* and a spherical SQD with the vacuum ground state |0〉 and the α-exciton state |ex〉 in the presence of an external field $E_{0}=Ee^{-i\omega t}+c.c.$. The SQD is placed in the gap of the nanoparticle dimer. The center-to-center distance between MNP a (MNP b) and the SQD is $d_{a}$ ($d_{b}$). The entire system is embedded in a dielectric medium with dielectric constant $\varepsilon_{0}$, as shown in Figure 13a.

The Hamiltonian of the SQD can be written as: $H_{QD}=\hbar \omega_{ex}\sigma_{z}$, where $\sigma_{z}=(|ex\rangle \langle ex|-|0\rangle \langle 0|)/2$. The plasmon field produced in MNPs can be quantized as a multi-modes field [14,18,19] $H_{M}=\hbar \sum_{k}\omega_{k}\left(a_{k}^{+}a_{k}+b_{k}^{+}b_{k}\right)$, where $\omega_{k}$ is the frequency of mode *k*, $a_{k}$ ($b_{k}$) is the annihilation operator of mode *k* in the MNP a (b). If the distance between the two MNPs is very large compared to the radius *R*, we can neglect the coupling between them, and only consider the coupling between the SQD and them because of the strong exciton-plasmon interaction [19]. Based on cavity quantum electrodynamics, the SQD can interact with the plasmon modes via the exchange energy. Under the rotating-wave approximation the interaction Hamiltonian between the SQD and the plasmon modes of the two MNPs can be written as [11,19] $H_{int}=-\hbar \sum_{k}\left[\left(g_{k}^{a}a_{k}+g_{k}^{b}b_{k}\right)\sigma_{+}+h.c.\right]$, where $\sigma_{+}=\left|ex\rangle \langle 0\right|$, $g_{k}^{a}$ ($g_{k}^{b}$) is the coupling constant of the SQD and the mode *k* in MNP a (b). In the excitation of an external field, the driving Hamiltonian is given by $H_{ext}=-\left\{\left[\left(\mu /\varepsilon_{eff}\right)\sigma +\sum_{k}\left(\mu_{k}^{a}a_{k}+\mu_{k}^{b}b_{k}\right)\right]Ee^{i\omega t}+h.c.\right\}$, where μ ($\mu_{k}^{a},\mu_{k}^{b}$) is the dipole moment between the ground state and the excited state |ex〉 of the SQD (the excited plasmon state ${|k\rangle }_{L}$ of MNP *L*, L=a,b), $\varepsilon_{eff}=\left(\varepsilon_{s}+2\varepsilon_{0}\right)/3\varepsilon_{0}$ is the screening factor with $\varepsilon_{s}$ being the dielectric constant of the SQD. So, the total Hamiltonian can be written as

(53) $$H=H_{QD}+H_{M}+H_{int}+H_{ext}$$

The full quantum dynamics of the hybrid molecule can be derived from the following master equation for the density operator

(54) $$i\hbar \partial_{t}\rho =\left[H,\rho \right]+i\hbar \left(\varsigma_{QD}+\varsigma_{a}+\varsigma_{b}\right),$$

with the Liouvillian terms $\varsigma_{QD}=\left(\kappa /2\right)\times \left(2\sigma \rho \sigma_{+}-\sigma_{+}\sigma \rho -\rho \sigma_{+}\sigma \right)$, $\varsigma_{L}=\sum_{k}\left(\gamma_{k}/2\right)\times \left(2L_{k}\rho L_{k}^{+}-L_{k}^{+}L_{k}\rho -\rho L_{k}^{+}L_{k}\right)$ describe the various scattering channels of molecule decay, plasmon decay through Laudau damping, and radiative decay [19].

Now, we define two bosonic operators $A_{k},B_{k}$ which satisfy the following linear relations:

(55) $$\alpha_{k}A_{k}=g_{k}^{a}a_{k}+g_{k}^{b}b_{k},\beta_{k}^{*}B_{k}={\left(g_{k}^{b}\right)}^{*}a_{k}-{\left(g_{k}^{a}\right)}^{*}b_{k},$$

where $|\alpha_{k}{|}^{2}=|\beta_{k}{|}^{2}=|g_{k}^{a}{|}^{2}+{\left|g_{k}^{b}\right|}^{2}$. We can see $\left[A_{k},A_{l}^{+}\right]=\left[B_{k},B_{l}^{+}\right]=\delta_{kl}$, $\left[A_{k},B_{l}^{+}\right]=\left[A_{k},B_{l}\right]=0$. Therefore, the total Hamiltonian of the hybrid molecule can be rewritten as $H=H_{Q}+H_{A}+H_{B}$, where $H_{Q}=\hbar \omega_{ex}\sigma_{z}-\left\{\left(\mu /\varepsilon_{eff}\right)\sigma Ee^{i\omega t}+h.c.\right\}$, $H_{A}=\hbar \sum_{k}\omega_{k}A_{k}^{+}A_{k}-\hbar \sum_{k}\left(\alpha_{k}A_{k}\sigma_{+}+\alpha_{k}^{*}A_{k}^{+}\sigma \right)-\left\{\sum_{k}\left[\left(\mu_{k}^{a}g_{k}^{a*}+\mu_{k}^{b}g_{k}^{b*}\right)/\alpha_{k}^{*}\right]A_{k}Ee^{i\omega t}+h.c.\right\}$, $H_{B}=\hbar \sum_{k}\omega_{k}B_{k}^{+}B_{k}-\left\{\left[\left(\mu_{k}^{a}g_{k}^{b}-\mu_{k}^{b}g_{k}^{a}\right)/\beta_{k}\right]B_{k}Ee^{i\omega t}+h.c.\right\}$. From the above Hamiltonian, the bosonic system $B_{k}$ can be considered an individual system because it does not interact with both the SQD and the bosonic system $A_{k}$. Thus, we only need to deal with the interaction between the bosonic system $A_{k}$ and the SQD.

The energy transfer between SQD and MNPs occurs in the hybrid molecule [5,61,62]. Strong coupling between SQD and two MNPs can give rise to the change of optical properties of SQD. For studying optical properties of SQD, we need to obtain the reduced density operator of the SQD. We now take a time-independent unity transformation $e^{s}$, $s=\sum_{k}\left[\pi_{k}^{*}\left(\omega_{ex}\right)A_{k}^{+}\sigma -\pi_{k}\left(\omega_{ex}\right)A_{k}\sigma_{+}\right]$, $\pi_{k}\left(\omega_{ex}\right)=\alpha_{k}/\left[\omega_{k}-\omega_{ex}-i\left(\gamma_{k}/2\right)\right]$, on the density operator ρ so that $\tilde{\rho }=e^{-s}\rho e^{s}$. Combining with Equation (54), we have $i\hbar \partial_{t}\tilde{\rho }=\left[e^{-s}He^{s},\tilde{\rho }\right]+i\hbar e^{-s}\left(\varsigma_{QD}+\varsigma_{a}+\varsigma_{b}\right)e^{s}$. In the mathematical expansions of the above equation the coupling between the plasmon modes and the SQD mainly appear in the terms of order $O(g_{k}^{3})$ and higher. We can neglect these high order terms for obtaining the reduced density operator of the SQD $\rho_{Q}=Tr_{M}\left[\tilde{\rho }\right]$. We assume that the plasmon modes can be consider as a thermal reservoir and the reservoir variables are distributed in the uncorrelated thermal equilibrium mixture of states [33], $\langle L_{k}^{+}L_{l}\rangle ={\bar{n}}_{k}^{L}\delta_{k,l}$, where the thermal average boson number ${\left({\bar{n}}_{k}^{L}\right)}^{-1}=exp\left[\left(\hbar \omega_{k}\right)/\left(k_{B}T\right)\right]-1$, $k_{B}$ is the Boltzmann constant, and *T* is the temperature. At low temperature, ${\bar{n}}_{k}^{L}\ll 1$. So, the master equation of the SQD can be written as (ℏ=1)

(56) $$\partial_{t}\rho_{Q}=-i\left[H_{Q},\rho_{Q}\right]+\varsigma_{Q}$$

where

(57) $$H_{Q}=\omega_{0}\sigma_{z}+\left(\mu_{0}E\sigma e^{i\omega t}+c.c.\right),$$

(58) $$\varsigma_{Q}=\left(\kappa_{0}/2\right)\left(2\sigma \rho_{Q}\sigma_{+}-\sigma_{+}\sigma \rho_{Q}-\rho_{Q}\sigma_{+}\sigma \right),$$

$\omega_{0}=\omega_{ex}-\sum_{k}\left[\alpha_{k}\pi_{k}^{*}\left(\omega_{ex}\right)+\alpha_{k}^{*}\pi_{k}\left(\omega_{ex}\right)\right]/2$ shows the shifted exciton frequency, $\kappa_{0}=\kappa +\sum_{k}\left[\alpha_{k}\pi_{k}^{*}\left(\omega_{ex}\right)-\alpha_{k}^{*}\pi_{k}\left(\omega_{ex}\right)\right]$ represents the modified exciton decay rate, and $\mu_{0}=\left(\mu /\varepsilon_{eff}\right)+\sum_{k}\left(\mu_{k}^{a}g_{k}^{a*}+\mu_{k}^{b}g_{k}^{b*}\right)/\left[\omega_{k}-\omega_{ex}-i\left(\gamma_{k}/2\right)\right]$. It is well known that the parameter $g_{k}^{a}\left(g_{k}^{b}\right)$ is related to the distance $d_{a}\left(d_{b}\right)$, and $\mu_{k}^{a}=\mu_{k}^{b}=\mu_{k}$ because of the identical MNPs. Based on the quantum-semiclassical correspondence [11,25,26,27], we have $\sum_{k}{\left|g_{k}^{L}\right|}^{2}/\left[\omega_{k}-\omega_{ex}-i\left(\gamma_{k}/2\right)\right]=\gamma \left(\omega_{ex}\right){\left(\mu S_{\alpha }\right)}^{2}R^{3}/\left(\hbar \varepsilon_{0}\varepsilon_{eff}^{2}d_{L}^{6}\right)$, $\gamma \left(\omega_{ex}\right)=\left[\varepsilon_{M}\left(\omega_{ex}\right)-\varepsilon_{0}\right]/\left[\varepsilon_{M}\left(\omega_{ex}\right)+2\varepsilon_{0}\right]$ and $g_{k}^{L}=\theta_{L}\mu_{k},$ where $\theta_{L}=\mu s_{\alpha }/\left(\hbar \varepsilon_{0}\varepsilon_{eff}d_{L}^{3}\right)$ is the real number. So, we can obtain $\omega_{0}=\omega_{ex}-Re\left[G\left(\omega_{ex}\right)\right]$, $\mu_{0}=\left(\mu /\varepsilon_{eff}\right)+C\left(\omega_{ex}\right)$, $\kappa_{0}=\kappa +\kappa_{nr,metal}$, $\kappa_{nr,metal}=2Im\left[G\left(\omega_{ex}\right)\right]$, where $G\left(\omega_{ex}\right)=\gamma \left(\omega_{ex}\right){\left(\mu S_{\alpha }\right)}^{2}R^{3}\left(d_{a}^{-6}+d_{b}^{-6}\right)/\left(\hbar \varepsilon_{0}\varepsilon_{eff}^{2}\right)$, $C\left(\omega_{ex}\right)=\gamma \left(\omega_{ex}\right)\mu S_{\alpha }R^{3}\left(d_{a}^{-3}+d_{b}^{-3}\right)/\varepsilon_{eff}$.

**Figure 14:**
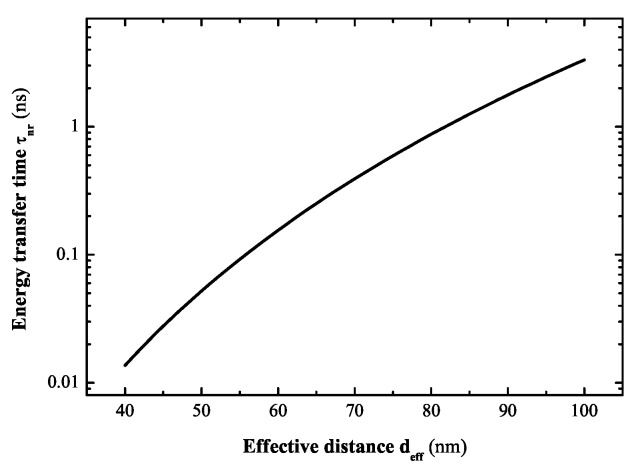
The energy transfer time between the QD and the two MNPs as a function of the effective distance $d_{eff}$.

We note that $\kappa_{nr,metal}=\left[2{\left(\mu S_{\alpha }\right)}^{2}R^{3}/\left(\hbar \varepsilon_{0}\varepsilon_{eff}^{2}d_{eff}^{6}\right)\right]\times Im\left[\gamma \left(\omega_{ex}\right)\right]$ represents the non-radiative decay rate of SQD as a result of the exciton-plasmon interaction, where the effective distance between the SQD and the two MNPs $d_{eff}^{-6}=d_{a}^{-6}+d_{b}^{-6}$. And the energy transfer time between the SQD and the MNPs can be defined as $\tau_{nr}=1/\kappa_{nr,metal}$ [70]. With the increase of the effective distance $d_{eff}$, the energy transfer time becomes longer. In what follows, we consider two identical Au nanoparticles with radius R=15 nm and a CdTe QD with r=3.75 nm, $\varepsilon_{s}=7.2$, the exciton energy $\hbar \omega_{ex}=2.5$ eV, and the exciton lifetime $\tau_{0}=20$ ns [1]. The dielectric constant of gold is ${\varepsilon_{M}\left(\omega \right)=\varepsilon }_{b}-\omega_{p}^{2}/\left[\omega \left(\omega +i\eta \right)\right]$ with $\varepsilon_{b}=9.5$, $\hbar \omega_{p}=9$ eV, ℏη=0.07 eV [36]. We take $\varepsilon_{0}=2$ (polymer), μ=0.65*e* nm. The exciton-dipole orientation parallel to the axis of the hybrid molecule $s_{\alpha }=2$. In Ref. [70], Govorov and co-authors illustrate that the dipole approximation is in agreement with the exact approach for the energy transfer time when the distance $d_{L}>3R$ (L=a,b). Under the dipole approximation, here in Figure 14, we plot the energy transfer time $\tau_{nr}$ as a function of the effective distance $d_{eff}$ in the hybrid molecule. We can see that the energy transfer time between the SQD and the two MNPs depends strongly on the effective distance between them. The effective distance includes two distances, $d_{a}$ and $d_{b}$.

The optical Bloch equations of the SQD are given by

(59) $$\partial_{t}\rho_{2,2}=-2Im\left[\mu_{0}E\rho_{2,1}\right]-\kappa_{0}\rho_{2,2},$$

(60) $$\partial_{t}\rho_{2,1}=i\left(\omega_{0}-\omega +i\kappa_{0}/2\right)\rho_{2,1}+i\mu_{0}^{*}E^{*}\left(2\rho_{2,2}-1\right),$$

where $\rho_{2,2}=\langle ex|\rho_{Q}|ex\rangle ,\rho_{2,1}=\langle ex|\rho_{Q}|0\rangle e^{i\omega t}$. According to the above equations, we can obtain the steady-state solutions

(61) $$\rho_{2,2}=\frac{|\mu_{0}{E|}^{2}}{{\left(\omega_{0}-\omega \right)}^{2}+\kappa_{0}^{2}/4+2{\left|\mu_{0}E\right|}^{2}},$$

(62) $$\rho_{2,1}=\frac{\left(\omega_{0}-\omega -i\kappa_{0}/2\right)\mu_{0}^{*}E^{*}}{{\left(\omega_{0}-\omega \right)}^{2}+\kappa_{0}^{2}/4+2{\left|\mu_{0}E\right|}^{2}}.$$

**Figure 15:**
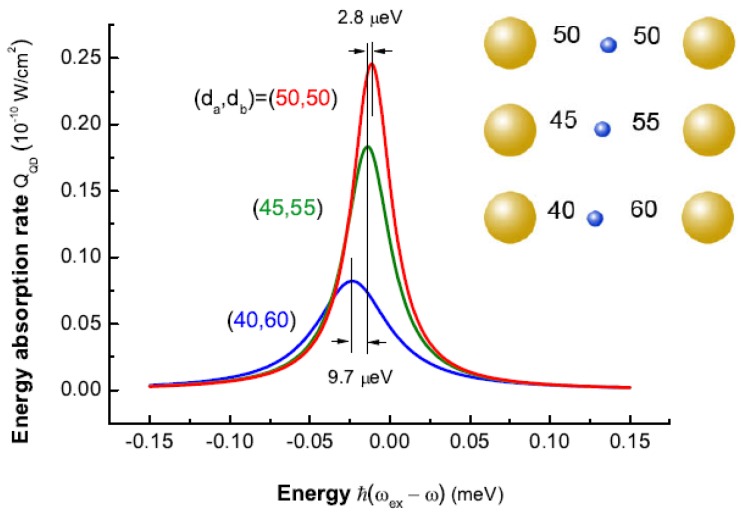
The energy absorption rate of the QD as a function of the energy difference $\hbar (\omega_{ex}-\omega )$ for different positions of the QD in the gap of the nanoparticle dimer in the excitation of a strong external field $I_{0}=$1000 W/cm${}^{2}$.

The SQD absorbs energy by the creation of an exciton. The absorbed energy comes from three channels, i.e., the external field and the two MNPs (MNP a and b). The energy absorption of the SQD depends strongly on the distances between the SQD and two MNPs. Thus, it is related to the position of the SQD in the gap of the nanoparticle dimer. The energy absorption rate of the SQD is given by $Q_{QD}=\hbar \omega_{ex}\rho_{2,2}/\tau_{0}$. Figure 15 plots the energy absorption rate of the SQD as a function of the energy difference $\hbar (\omega_{ex}-\omega )$ for the distances $\left(d_{a},d_{b}\right)$ in a strong external field (the intensity is $I_{0}=$ 1000 W/cm${}^{2}$). We can see that the energy absorption peak represents the exciton energy shift because the exciton-plasmon interaction can give rise to the modification of the exciton energy. In many reports, authors have investigated an ideal theoretical model in which the distances between SQD and two MNPs are equal ($d_{a}=d_{b}$) [60,71,72]. Here, if the distances $d_{a}=d_{b}=50$ nm are chosen in our model, an exciton energy shift about 11 μeV is shown in the energy absorption spectrum of the QD. Considering the real distances in experiments, however, we need to study the exciton energy shift under the condition $d_{a}\neq d_{b}$. If the QD make a movement 5 nm to the direction of the MNP a, the exciton energy shift has increase by 2.8 μeV based on our theory. Let the QD does this again, the shift can reach 23.5 μeV. Thus, the position of the QD in the gap of the nanoparticle dimer is important for the modification of the exciton energy as a result of the exciton-plasmon interaction.

In the presence of a strong external field, the energy absorption rate of every MNP at Fano resonance is larger than that of SQD. So, the energy absorption of the two MNPs is significant for the total energy absorption of the molecule. The exciton energy, in the hybrid molecule, can be transferred from SQD to MNPs, and then converted into heat [3,6]. The energy absorption rate of the MNPs $Q_{M}=\sum_{k}\hbar \omega_{k}\gamma_{k}\left(\langle a_{k}^{+}a_{k}\rangle +\langle b_{k}^{+}b_{k}\rangle \right)/2=\sum_{k}\hbar \omega_{k}\gamma_{k}\left(\langle A_{k}^{+}A_{k}\rangle +\langle B_{k}^{+}B_{k}\rangle \right)/2$. Combining Equations (54) and (61), we can obtain the steady-state solutions of $\langle A_{k}^{+}A_{k}\rangle$ and $\langle B_{k}^{+}B_{k}\rangle$. The total energy absorption rate of the two MNPs including two parts, can be written as $Q_{M}=Q_{a}+Q_{b},$ $Q_{a}$ ($Q_{b}$) represents the energy absorption rate of MNP a (b), where

(63) $$Q_{L}=Q_{0}\times \frac{{\left(\Delta -M_{L}\right)}^{2}+N_{L}^{2}}{\Delta^{2}+1},\left(L=a,b\right)$$

$Q_{0}=3\omega \varepsilon_{0}^{2}Im\left[\varepsilon_{M}\left(\omega \right)\right]R^{3}E^{2}/\left[2\right|\varepsilon_{M}\left(\omega \right)+2\varepsilon_{0}{|}^{2}]$, $\Delta =\left(\omega_{0}-\omega \right)/\sqrt{\kappa_{0}^{2}/4+2{\left|\mu_{0}E\right|}^{2}}$, $M_{L}=Re\left[\mu_{0}\right]\theta_{L}/\sqrt{\kappa_{0}^{2}/4+2{\left|\mu_{0}E\right|}^{2}}$, $N_{L}=\sqrt{{\left(\kappa_{0}/2-\theta_{L}Im\left[\mu_{0}\right]\right)}^{2}+2{\left|\mu_{0}E\right|}^{2}}/\sqrt{\kappa_{0}^{2}/4+2{\left|\mu_{0}E\right|}^{2}}$. The expression of the energy absorption rate shows a Fano function [23] with the generalized field–dependent complex Fano factor which includes both the nonlinear and dephasing effects [15]. The exciton-plasmon interaction gives rise to Fano interference process in the coupled SQD-MNP system. The excitation of the plasmon field in MNP can be implemented by two competing optical pathways which causes Fano interference.

**Figure 16:**
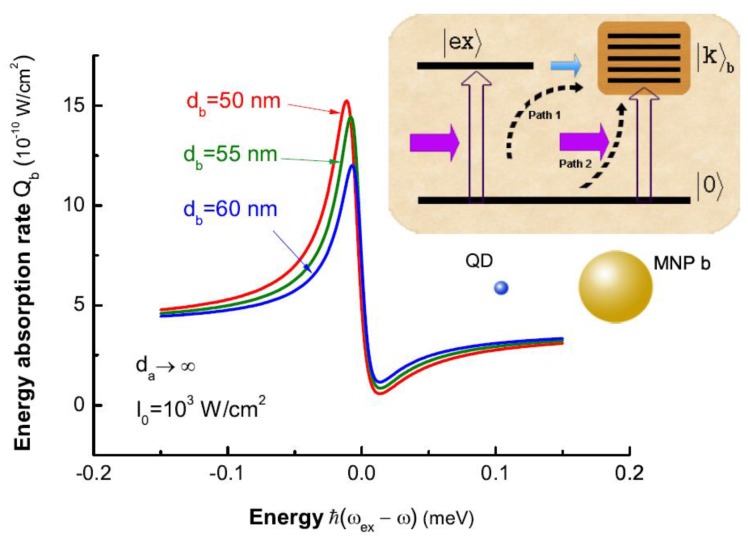
The energy absorption rate of MNP b $Q_{b}$ as a function of the energy difference $\hbar (\omega_{ex}-\omega )$ for the distances $d_{b}=50,55,60$ nm in the absence of MNP a in strong external field $I_{0}=$ 1000 W/cm${}^{2}$. Inset: Quantum interference pathways (path 1 and 2) to excite the plasmon states ${|k\rangle }_{b}$ in the steady-state limit.

In a molecule including a SQD and a MNP, Fano effect was reported because of the exciton-plasmon interaction [11]. In the absence of MNP a ($d_{a}\to \infty$), in our theory, Fano interference process can also occur under the excitation of the external field. Fano interference refers to three states [24], i.e., the common ground state |0〉, an excited state |ex〉 and an infinite collection of plasmon states ${|k\rangle }_{b}$. As shown in inset of Figure 16, there are two optical excitation transitions (|0〉→|ex〉 and $|0\rangle \to {|k\rangle }_{b}$) and one coupling transition ($|ex\rangle \to {|k\rangle }_{b}$). Two optical pathways (path 1 and 2 in the inset of Figure 15) can be found to generate the states ${|k\rangle }_{b}$ . Constructive or destructive interference between two pathways, depending on the energy difference between the exciton and the external field, give rise to the Fano effect. Using Equation (62), the energy transfer rate of MNP b can be written as $Q_{b}=Q_{0}\times \left[{\left(\Delta -M_{b}\right)}^{2}+N_{b}^{2}\right]/\left[\Delta^{2}+1\right].$ In Figure 16, we illustrate the energy absorption rate of MNP b $Q_{b}$ as a function of energy difference $\hbar (\omega_{ex}-\omega )$ for the distances $d_{b}=50,55,60$ nm in the absence of MNP a ($d_{a}\to \infty$). The asymmetric Fano profile shown in the energy absorption spectrum is caused by Fano interference process of optical pathways in the inset of Figure 16.

**Figure 17:**
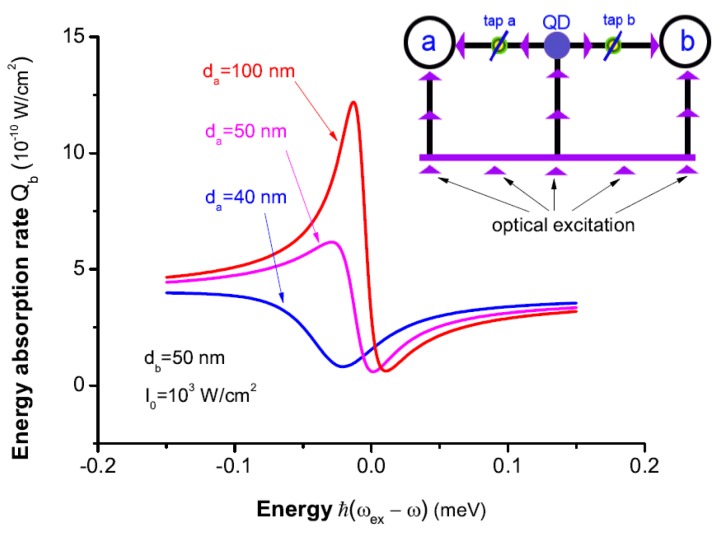
The energy absorption rates of MNP b $Q_{b}$ ($d_{b}=50$ nm) as a function of the energy difference $\hbar (\omega_{ex}-\omega )$ for the distances $d_{a}=100,50,40$ nm. Inset shows all optical pathways of two correlated Fano interference processes in optical excitation.

In the simultaneous presence of MNP a and b, there are two correlated Fano interference processes. In Figure 13b, we can see that the two processes share a common segment of optical pathway (|0〉→|ex〉) between them. And the coupling of the SQD to the two MNPs determine the quantum amplitudes of the optical pathways ($|0\rangle \to |ex\rangle \to {|k\rangle }_{a}$ and $|0\rangle \to |ex\rangle \to {|k\rangle }_{b}$), to affect the excitations of ${|k\rangle }_{a}$ and ${|k\rangle }_{b}$. In other words, as shown in the inset of Figure 17, the coupling of SQD to MNP seems a “tap” of optical pathway to excite the plasmon states of the MNP. Because the coupling is related to the distance between them, “tap” can be controlled by the corresponding distance. In all optical pathways, there are two “taps” which can affect the excitation of the plasmon states of every MNP all together. So, the energy absorption rate of one MNP not only depends on the distance between itself and SQD (see Figure 16), but also it is related to the distance between another MNP and SQD. This is because of the correlation of two Fano interference processes. To clearly illustrate the interesting correlation of two Fano interference processes, we plot the energy absorption rates of MNP b $Q_{b}$ ($d_{b}=50$ nm) for the distances $d_{a}=100,50,40$ nm. In Figure 17, we can see that the distance between MNP a and the SQD plays an important role in the energy absorption rate of MNP b. By adjusting the distance $d_{a}$ (just as manipulating tap a in the inset of Figure 17), we can change the energy absorption of MNP b. In our theory, so, controlling the position of one MNP is a potential approach to change the energy absorption of another MNP because of the correlation.

We have defined a effective distance $d_{eff}$ for conveniently studying the energy transfer time between SQD and two MNPs. The effective distance is important for SQD, because it determines the shifted exciton energy $\omega_{0}$ and the energy transfer time $\tau_{nr}$. For obtaining the total energy absorption, however, we need to define a correlated distance $d_{c}$ which satisfies $d_{c}^{-3}=d_{a}^{-3}+d_{b}^{-3}$, and $d_{c}\in \left[2^{-1/6}d_{eff},d_{eff}\right].$ It can show the correlation of two correlated Fano interference processes for a given energy transfer time.

## 6. Conclusions

Based on cavity quantum electrodynamics, we have investigated the light-matter interaction between metal nanoparticles (MNPs) and semiconductor quantum dots (SQDs) in a hybrid SQD-MNP system. By using quantum transformation method, we reveal that the quantized plasmon field in MNPs gives rise to the exciton energy shift and the modified decay rate of SQDs near the MNPs. In a hybrid molecule including a SQD and a MNP, we study the optical response of the hybrid molecule for one or two external fields. Fano effect can be observed in the absorption spectrum of the MNP, which originates from constructive or destructive interference between two optical pathways. A tunable optical response scheme is proposed, which can be potentially applied in optical processing devices. We also show quantum entanglement of two SQDs induced by the quantization plasmon field of a MNP in a hybrid molecule including two SQDs and a MNP. Quantum entanglement shows the quantum correlations of quantum systems which can be exhibited by the violation of some inequalities, such as Leggett-Garg inequalities for a single system [73], Bell inequalities for multiple spatially separated systems [74]. Concurrence can quantify entanglement between two quantum systems, which is proposed by Wootters [55]. In the excitation of an external field, the steady-state entanglement of the two SQDs can be implemented, and its concurrence can be obtained by means of the observation of Fano profile shown in the absorption spectrum of the hybrid molecule. We also study the coupling of a SQD to two MNPs. In the hybrid system, it is shown that a Fano correlation effect shown in the energy absorption spectrum appears, which stems from two correlated Fano interference processes because the two MNPs share a common segment of optical pathway involving SQD as a result of the plasmon-exciton-plasmon interaction. Our results suggested that the spatial structure of a complex SQD-MNP system determines quantum nature of the exciton-plasmon interaction, which can be revealed by observing its optical properties. Therefore, the investigation of quantum description for complex SQD-MNP system provides a bridge between the spatial structure and the observed optical phenomena.
